# Going faster to see further: graphics processing unit-accelerated value iteration and simulation for perishable inventory control using JAX

**DOI:** 10.1007/s10479-025-06551-6

**Published:** 2025-03-24

**Authors:** Joseph Farrington, Wai Keong Wong, Kezhi Li, Martin Utley

**Affiliations:** 1 Institute of Health Informatics, University College London, London, UK; 2 NIHR University College London Hospitals Biomedical Research Centre, University College London, London, UK; 3 University College London Hospitals NHS Foundation Trust, London, UK; 4 Cambridge University Hospitals NHS Foundation Trust, Cambridge, UK; 5 Clinical Operational Research Unit, University College London, London, UK

**Keywords:** Inventory, Markov decision processes, Dynamic programming, Simulation, Reinforcement learning

## Abstract

Value iteration can find the optimal replenishment policy for a perishable inventory problem, but is computationally demanding due to the large state spaces that are required to represent the age profile of stock. The parallel processing capabilities of modern graphics processing units (GPUs) can reduce the wall time required to run value iteration by updating many states simultaneously. The adoption of GPU-accelerated approaches has been limited in operational research relative to other fields like machine learning, in which new software frameworks have made GPU programming widely accessible. We used the Python library JAX to implement value iteration and simulators of the underlying Markov decision processes in a high-level interface, and relied on this library’s function transformations and compiler to efficiently utilize GPU hardware. Our method can extend use of value iteration to settings that were previously considered infeasible or impractical. We demonstrate this on example scenarios from three recent studies which include problems with over 16 million states and additional problem features, such as substitution between products, that increase computational complexity. We compare the performance of the optimal replenishment policies to heuristic policies, fitted using simulation optimization in JAX which allowed the parallel evaluation of multiple candidate policy parameters on thousands of simulated years. The heuristic policies gave a maximum optimality gap of 2.49%. Our general approach may be applicable to a wide range of problems in operational research that would benefit from large-scale parallel computation on consumer-grade GPU hardware.

**Supplementary Information:**

The online version contains supplementary material available at 10.1007/s10479-025-06551-6.

## Introduction

Perishable items, such as fresh food and blood products, “*undergo change in storage so that in time they may become partially or entirely unfit for consumption*” (Nahmias, 1982). This means that wastage must be considered alongside the impact of shortages and stock-holding levels when making replenishment decisions. Wastage may be a concern for economic, sustainability, or ethical reasons. One of the United Nations Sustainable Development Goals is to halve the estimated 17% wastage at the retail and consumer levels of the food supply chain (United Nations, 2022). In the blood supply chain, platelets can only be stored for between 3 and 7 days leading to high reported wastage rates of 10–20% (Flint et al., 2020). Better policies for perishable inventory control could help to reduce wastage.

Early theoretical work demonstrated that optimal policies for perishable inventory replenishment could be found using dynamic programming (Nahmias, 1975b; Fries, 1975). Value iteration is a dynamic programming approach that can be used to find the optimal policy for problems framed as a Markov decision process (MDP) (Bellman, 1957). The optimal policy depends on the age profile of the inventory and is therefore limited by the “curse of dimensionality”: the computational requirements grow exponentially with the maximum useful life of the product (Nahmias, 2011). Nahmias (1982) observed that this (then) made dynamic programming approaches impractical for problems where the maximum useful life of the product was more than two periods. More recently, despite advances in computational power, researchers have stated that value iteration remains infeasible or impractical when the maximum useful life of the product is longer than two or three periods or when additional complexities (e.g. substitution between products or random remaining useful life on arrival) are introduced (De Moor et al., 2022; Hendrix et al., 2019; Mirjalili, 2022).

The prevalent view in the perishable inventory literature about the scale of problems for which value iteration is feasible appears to neglect recent developments in graphics processing unit (GPU) hardware, and in software libraries that make it possible for more researchers and practitioners to take advantage of GPU capabilities. GPUs were developed for rendering computer graphics, which requires the same operations to be efficiently applied to many inputs in parallel. Compared to a central processing unit (CPU), GPUs therefore have many more, albeit individually less powerful, cores. GPU-acceleration refers to offloading computationally intensive tasks that benefit from large-scale parallelization from the CPU to GPUs. The first mainstream software framework to support general computing tasks on GPUs using a common general purpose programming language was Nvidia’s Compute Unified Device Architecture (CUDA) platform, which launched in 2007. One of the areas in which GPUs have since had a major impact is the field of deep learning. This impact has led to, and in turn been supported by (Jeon et al., 2021), the development of higher-level software libraries including Numba (Lam et al., 2015), TensorFlow (Abadi et al., 2016), PyTorch (Paszke et al., 2019) and JAX^1^ (Bradbury et al., 2022). These libraries provide comparatively simple Python application programming interfaces (APIs) to support developers while utilising highly optimized CUDA code “under-the-hood” to exploit the parallel processing capabilities of GPUs.

In this work, we implemented value iteration using JAX. JAX provides composable transformations of Python functions, which make it easy to apply functions in parallel over arrays of input data, and performs just-in-time compilation (JIT) to run workloads efficiently on hardware accelerators including GPUs. This is ideal for value iteration: each iteration requires many independent updates which can be performed in parallel, and the up-front computational cost of JIT can be amortised over many repeats of the compiled operation for each iteration. By decreasing the wall time (the real, elapsed time required for a program to run measured by the computer’s clock) required to run value iteration, we increase the size of problems for which the optimal policy can be calculated in practice: by going faster, we can see further. These policies can directly influence decision making. They can also support research into new heuristics and approximate approaches, including reinforcement learning, by providing performance benchmarks for much larger problems than has previously been possible.

Inspired by the recent development of GPU-based simulators in reinforcement learning (Freeman et al., 2021; Makoviychuk et al., 2021; Lange, 2022b; Bonnet et al., 2024), we also implemented simulators for perishable inventory problems using JAX, enabling an extensive search of possible parameters for heuristic policies, and small sampling errors when evaluating policies.

We consider perishable inventory scenarios from three recent studies where running value iteration was described as computationally infeasible or impractical (De Moor et al., 2022; Hendrix et al., 2019; Mirjalili, 2022). For all but the most challenging setting (with over 12.6 billion states), we found the optimal policy using a consumer-grade GPU and report the wall time required. We compare the performance of heuristic policies with parameters fitted using simulation optimization to performance of the policies found using value iteration.

The main contributions of our work are:

- demonstrating that value iteration can be used for perishable inventory replenishment problems for which it was recently described as computationally infeasible or impractical, using consumer-grade GPU hardware (summarised in Table 1);
- providing an open-source, general purpose implementation of value iteration for MDPs using the Python library JAX, and three examples of how it can be customised to solve specific problems without in-depth knowledge of GPU-specific programming approaches and frameworks;
- demonstrating that simulation optimization can also be run in parallel effectively using JAX, such that good policies can be identified for larger problems in a fraction of the time required to run value iteration.

For one problem with over 16 million states (Hendrix et al., 2019), a CPU-based MATLAB implementation of value iteration did not converge within a week in a prior study. Using our method, value iteration converges in under 3.5 h on a consumer-grade GPU and, without any code changes, in less than 30 min using four data-centre grade GPUs. Our simulation optimization method is able to evaluate 50 possible sets of parameters for a heuristic policy, each on 4000 years of simulated data, in parallel in under 15 s. The largest optimality gap we observed for the heuristic policies fit using our parallel implementation of simulation optimization was 2.5%.

Our code is available at https://github.com/joefarrington/viso_jax. The repository includes a Google Colab notebook that enables interested readers to reproduce our experiments using free cloud-based GPUs provided by Google.

**Table 1:** Summary of our contribution extending value iteration to larger problems with a longer maximum useful life

		Maximum useful life *m*				
	Problem features	2	3	4	5	8
A	Lead time > 1					
B	Substitution between products					
C	Not all arrivals fresh, periodic demand					

Key:

Value iteration feasible for all experiments in the original study

Our method extends value iteration to experiments that were considered infeasible or impractical in the original study

All experiments infeasible in the original study and with our method

Setting not considered in the original study

## Related work

Diverse sequential decision making problems can be modelled as MDPs and solved using value iteration. Jóhannsson (2009) demonstrated that value iteration could be effectively run in parallel on a GPU soon after the introduction of CUDA. Subsequent research has evaluated the performance of GPU-accelerated value iteration on problems from economics and finance (Aldrich et al., 2011; Aamer et al., 2020; Duarte et al., 2020; Kirkby, 2017, 2022) and route-finding and navigation (Chen & Lu, 2013; Inamoto et al., 2011; Ruiz & Hernández, 2015; Constantinescu et al., 2020). We have only identified a single study that applied this approach to an inventory control problem: Ortega et al. (2019) implemented a custom value iteration algorithm in CUDA to find replenishment policies for a subset of perishable inventory problems originally described by Hendrix et al. (2019).

Previous studies have reported impressive reductions in wall time achieved by running value iteration on GPU. The GPU-accelerated method in Ortega et al. (2019) was up to 11.7$\times$ faster than a sequential CPU-based method written in C. Despite this, the approach has not been widely adopted. The aforementioned studies focus predominantly on implementing value iteration using CUDA (or OpenCL, a multi-platform alternative) and comparing the performance of a GPU-accelerated method with a CPU-based method, instead of seeking to use GPU-acceleration to solve problems for which value iteration is otherwise impractical or infeasible. A similar approach has been used to develop GPU-accelerated methods for other operational research problems (Yianni et al., 2018; Bäumelt et al., 2016; Boschetti et al., 2017). One of the main barriers to entry for some researchers is the perceived difficulty of GPU programming. Writing efficient code using CUDA or OpenCL requires careful consideration of memory access, balancing resource usage when mapping parallel processes to the hardware, and interaction between the CPU and GPU (Hijma et al., 2022). There have been efforts to make GPU-accelerated value iteration more accessible. Jóhannsson (2009) created a solver framework using his CUDA implementation of value iteration as a back-end, but this does not appear to be publicly available. More recently, Kirkby (2017) created a toolkit in MATLAB to solve infinite horizon value iteration problems which automatically uses a GPU when available and appropriate but this requires the state-transition matrix to be provided as an input, which is infeasible for some problems we consider due to memory limitations.

Our approach is similar to that of Duarte et al. (2020) and Sargent and Stachurski (2022) who used machine learning frameworks to implement GPU-accelerated value iteration for economics models. A key observation made by Duarte et al. (2020) is that their TensorFlow implementation is an order of magnitude faster than their custom CUDA C++ implementation on a GPU. This demonstrates that the comparative accessibility provided by a machine learning framework need not incur poorer performance. TensorFlow, like PyTorch and JAX, translates the high level API instructions into highly-optimized CUDA code. Researchers without expertise in GPU programming are likely to save development time and achieve better performance by working at a higher level of abstraction using a machine learning framework and relying on it to make best use of the available hardware.

Due to the computational challenges of dynamic programming, previous work has focused on approximate solutions by down-sizing the problem (Blake et al., 2003; Haijema et al., 2007) or using heuristic policies with a small number of parameters, such as a base-stock policy. Research on heuristic policies has concentrated on identifying suitable policy structures (see Nahmias, 1975a for an early example, and Haijema & Minner, 2019 for a recent example), and finding suitable parameters for those policies in specific situations—commonly using stochastic mixed integer linear programming (Dillon et al., 2017; Gunpinar & Centeno, 2015; Rajendran & Ravindran, 2017) or simulation optimization (Dalalah et al., 2019; Duan & Liao, 2013). Recently, reinforcement learning methods have also been used to find approximate polices for managing perishable inventory (Kara & Dogan, 2018; Sun et al., 2019; De Moor et al., 2022; Ahmadi et al., 2022).

We focus on simulation optimization in addition to value iteration because GPU-accelerated simulation using available software libraries is not yet widely adopted. Applied research on specific mixed integer linear programs often relies on commercial solver software such as IBM ILOG CPLEX Optimization Studio or Gurobi Optimizer which do not currently support GPU-acceleration. Adapting mixed integer programming solution strategies to suit the architecture of GPUs is the subject of active research (Perumalla & Alam, 2021).

Simulation optimization can be used to solve optimization problems where the objective function cannot be computed exactly, but can be estimated using simulation. Sampling error in the objective function can be reduced by running simulations for a longer period, or by running additional simulations. The relevance of parallel computing to simulation optimization is well recognised (Amaran et al., 2016; Fu et al., 2014), but we have identified few simulation optimization studies using GPUs to run multiple simulations in parallel. In inventory management, Srimool et al. (2011) exhaustively evaluated the possible order quantities for a newsvendor problem using parallel simulations on GPU. More recently, Lau and Srinivasan (2016) used simulation optimization to solve a chemical process monitoring problem using GPU-acceleration for both the simulation and the metaheuristic search process that proposed candidate solutions. Both of these projects used custom CUDA code which may explain the limited adoption despite the established reductions in wall time. We implemented simulators using the Python library gymnax (Lange, 2022b), which enabled us to use JAX and readily evaluate each of numerous policies on thousands of simulated years in parallel on GPU.

## Methods

### Scenarios

We considered three periodic review, single-echelon perishable inventory problem scenarios with a fixed, known delivery lead time *L*. These were selected as recent examples from the perishable inventory literature in which value iteration was reported as infeasible or impractical for some experimental settings, and which include elements relevant to our ultimate goal of supporting blood product inventory management. Scenario A, from De Moor et al. (2022), is a straightforward perishable inventory replenishment problem but for some experimental settings the lead time, *L*, is greater than one period and therefore we need to consider inventory in transit when placing an order. Scenario B is the two product scenario described by Hendrix et al. (2019) which adds the complexity of substitution between perishable products. Substitution is an important aspect of managing blood product inventory, where compatibility between the blood groups of the donor and the recipient is critical. Scenario C, from Mirjalili (2022), models the management of platelets in a hospital blood bank and adds two complicating factors: periodic patterns of demand, and uncertainty in the remaining useful life of products on arrival, which may depend on the order quantity. In every scenario demand is stochastic, unmet demand is assumed to be lost, and units in stock with a remaining useful life of one period are assumed to expire at the end of the day. Except in Scenario C, the products have a fixed, known useful life *m* and are all assumed to arrive fresh. We summarise the key differences between the scenarios in Table 2.

For those unfamiliar with inventory management problems and the associated terminology we recommend Chapters 3 and 4 of Snyder and Shen (2019) for a general introduction and Chaudhary et al. (2018) and Nahmias (2011) for more focused coverage of perishable inventory control.

**Table 2:** Summary of the key differences between our three scenarios

		Problem features					Reward function components					
	Source	Products	Lead time $> 1$	Substitution	Not all arrivals fresh	Periodic demand	Variable ordering	Fixed ordering	Wastage	Shortage	Holding	Revenue
A	De Moor et al. (2022)	1	$\checkmark$				$\checkmark$		$\checkmark$	$\checkmark$	$\checkmark$	
B	Hendrix et al. (2019)	2		$\checkmark$			$\checkmark$					$\checkmark$
C	Mirjalili (2022)	1			$\checkmark$	$\checkmark$		$\checkmark$	$\checkmark$	$\checkmark$	$\checkmark$	

Each study used different notation. To highlight the similarities and differences between the scenarios we have adopted a single notation to describe all three scenarios. In Supplementary Information A.1, B.1 and C.1 we present the key equations describing the respective scenario in our notation and in Supplementary Information D we provide a table summarising our notation.

### Markov decision processes

All of the scenarios are defined as Markov decision processes. An MDP is a formal description of a sequential decision problem in which, at a discrete series of points in time, an agent observes the state of its environment $S_t$ and selects an action $A_t$. At the next point in time, the agent will receive a reward signal $R_{t+1}$, observe the updated state of its environment $S_{t+1}$ and must select its next action $A_{t+1}$. An MDP can be defined in terms of a set of states $s \in \mathbb {S}$, a set of actions $a \in \mathbb {A}$, a set of a rewards $r \in \mathbb {\Psi }$, a function defining the dynamics of the MDP (Eq. 1), and a discount factor $\gamma \in [0,1]$ (Sutton & Barto, 2018). The discount factor controls the relative contribution of future rewards and immediate rewards. The decision process is Markovian because the dynamics of the system obey the Markov property: state transitions and rewards at time *t* are conditionally independent of the sequence of state-action pairs $(S_0, A_0)$ to $(S_{t-1}, A_{t-1})$ given $(S_t, A_t)$.

1 $$\begin{aligned} p(s', r|s,a) = \text {Prob}\left( S_t=s', R_t=r|S_{t-1}=s, A_{t-1}=a\right) \end{aligned}$$

Within this framework, MDP agents select their actions by following a policy. In this work, we only consider deterministic policies, $a=\pi (s)$. The objective is to find a policy that maximises the expected return, the discounted sum of future rewards, when interacting with the environment. In an infinite horizon problem the return at timestep *t* is $G_t = \sum _{k=0}^{\infty } \gamma ^{k}R_{t+k+1}$.

### Value iteration

Following the treatment of Sutton and Barto (2018), the value of a state under a policy $\pi$, $V^{\pi }(s)$, is the expected return when starting in state *s* and following policy $\pi$. For an MDP in which the sets of states, actions and rewards are finite, we can define an optimal policy, $\pi ^*$, as a policy for which $V^{\pi ^*}(s) \ge V^{\pi '}(s)$ for every state $s \in \mathbb {S}$, for any policy $\pi '$. There may be multiple optimal policies, but they all share the same optimal value function. Value functions satisfy recursive relationships, called Bellman equations, between the value at the current state and the immediate reward plus the discounted value at the next state. The Bellman equation for the optimal policy, the Bellman optimality equation, is:

2 $$\begin{aligned} V^{\pi ^*}(s) = \max _{a \in \mathbb {A}} \sum _{s' \in \mathbb {S}, r \in \mathbb {\Psi }} p(s',r|s,a) \left[ r + \gamma V^{\pi ^*}(s')\right] \end{aligned}$$

Value iteration is a dynamic programming algorithm, which uses the Bellman optimality equation as an update operation to estimate the optimal value function:

3 $$\begin{aligned} V_{i+1}(s) = \max _{a \in \mathbb {A}} \sum _{s' \in \mathbb {S}, r \in \mathbb {\Psi }} p(s',r|s,a) \left[ r + \gamma V_i(s')\right] \end{aligned}$$

For such a finite MDP this operation will, in the limit of infinite iterations, converge to the optimal value function. The optimal policy can be extracted from the value function using a one-step ahead search:

4 $$\begin{aligned} \pi (s) = \mathop {\mathrm {arg\,max}}\limits _{a \in \mathbb {A}} \sum _{s' \in \mathbb {S}, r \in \mathbb {\Psi }} p(s',r|s,a) \left[ r + \gamma V(s')\right] \end{aligned}$$

### Implementing value iteration in JAX

In the scenarios considered in this study the state reflects the inventory position and other problem-dependent information, the actions are replenishment order quantities, and the reward reflects daily income and/or costs.

Similar to the approach of Hendrix et al. (2019), we used a deterministic transition function, $(s', r) = T(s, a, \omega )$, where $\omega \in \mathbb {\Omega }$ is a possible realisation of the stochastic element(s) of the transition. For a specific state-action pair, (*s*, *a*), and a specific random outcome, $\omega$, the next state and the reward can be calculated deterministically. In the most straightforward example we consider, Scenario A, the only uncertainty is in the daily demand, and therefore $\mathbb {\Omega }$ is the set of possible values that demand may take in any period. Under this formulation, the value iteration update equation can be rewritten as:

5 $$\begin{aligned} V_{i+1}(s) = \max _{a \in \mathbb {A}} \sum _{\omega \in \mathbb {\Omega }} P(\omega |s,a) \left[ r_{\omega } + \gamma V_i(s'_{\omega })\right] , \text {where } (r_{\omega }, s'_{\omega }) = T(s, a, \omega ) \end{aligned}$$

and we extract the optimal policy using the equation:

6 $$\begin{aligned} \pi (s) = \mathop {\mathrm {arg\,max}}\limits _{a \in \mathbb {A}} \sum _{\omega \in \mathbb {\Omega }} P(\omega |s,a) \left[ r_{\omega } + \gamma V(s'_{\omega })\right] , \text {where } (r_{\omega }, s'_{\omega }) = T(s, a, \omega ) \end{aligned}$$

where $P(\omega |s,a) = \text {Prob}\left( \Omega _t = \omega | S_t = s, A_t = a\right)$ is the probability of random outcome $\omega$ having observed state $S_t=s$ and then taken action $A_t=a$. $\Omega _t$ represents the stochastic elements of the transition that occur between the observation of state $S_t$ and the observation of state $S_{t+1}$.

Since we cannot run an infinite number of iterations, we use a convergence test to determine when to stop value iteration and extract the policy. We are interested in the policy, and not the value function itself, and therefore in certain cases we can reduce the number of iterations required by stopping when further updates to the value function will not change the policy. We describe the convergence test used for each scenario in the corresponding section below.

We implemented value iteration using a custom Python class, ValueIterationRunner, which defines the common functionality required to run value iteration and extract the optimal policy using the approach in Eqs. 5 and 6. For each scenario we defined a subclass of ValueIterationRunner with custom functions that:

- return a list of all possible states as tuples;
- return an array that maps from a state to its index in the list of all possible states;
- return an array of all possible actions;
- return an array of all possible random outcomes;
- return the immediate reward and next state following the deterministic transition function given a state, action and random outcome;
- return an array with the probability of each random outcome given a state-action pair;
- return an initial estimate of the value function; and
- test for convergence of the value iteration procedure.

A naive implementation of value iteration following Eq. 5 would require a nested for-loop over every state, every action, and every random outcome. Every iteration, *i*, requires an outer loop over every state but the updates for each state can be computed independently using the estimates of the value function from the previous iteration $i-1$. This independence means that, during each iteration, the value function updates for each state can be computed in parallel instead of in sequence. At the end of each iteration, the results of the parallel updates can be combined into a complete updated estimate of the value function, which is then distributed as an input for the next iteration. JAX provides two main composable function transformations that facilitate running functions in parallel: vectorizing map (vmap) and parallel map (pmap). Both of these transformations create new functions that map the original function over specified axes of the input, enabling the original function to be applied to a large number of inputs in parallel. The key difference is that vmap provides vectorization, so the operations happen on the same device (e.g. the same GPU), while pmap supports single-program, multiple-data parallelism and runs the operation for different inputs on separate (but identical) devices.

An important feature of vmap and pmap is that they are composable and therefore can be readily nested. For our basic value iteration update, set out in Algorithm 1, we nest vmap operations over states, actions, and random outcomes instead of using nested loops. This is only feasible if there is sufficient GPU memory to update all of the states simultaneously. For larger instances we grouped the states into batches for which the update can be performed simultaneously and performed the update for one batch of states at a time. To enable multiple identical devices to be used where available, we automatically detected the number of available devices and used pmap to map our update function for multiple batches of states over the available devices. Each batch must contain the same number of states, and each device must receive the same number of batches, to efficiently loop over batches of states and use pmap. We therefore padded the array of states so that it could be reshaped to an array with dimensions (*number of devices*, *number of batches*, *maximum batch size*, *number of elements in state*). Each device received an array with dimensions (*number of batches*, *maximum batch size*, *number of elements in state*), performed a loop over the leading dimension, and calculated the update one batch of states at a time. The same process was used to extract the policy in parallel at the end of value iteration.

**Algorithm 1:**
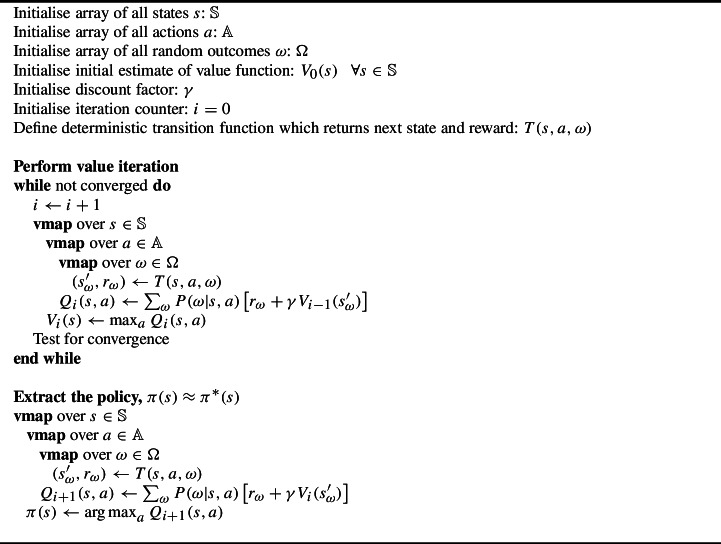
Value iteration using vmap

Functions transformed by pmap are automatically JIT compiled with XLA, a domain-specific compiler for linear algebra. JAX traces the function the first time it is run, and the traced function is compiled using XLA into optimized code for the available devices.

We used double-precision (64-bit) numbers when running value iteration, instead of the single-precision (32-bit) numbers that JAX uses by default because, in preliminary experiments with Scenario A which required a large number of iterations for convergence, we found that convergence was not always stable at 32-bit.

We report the wall time required to run each value iteration experiment. The reported times include JIT compilation time, writing checkpoints and writing final outputs, including the policy, because we believe this represents a realistic use case.

We focused on standard value iteration for consistency with the studies our scenarios were drawn from. However, alternative algorithms may offer further improvements in computational speed. One such alternative is asynchronous value iteration (Sutton & Barto, 2018), in which updates to the value function are immediately available for calculating the updated values of other states, rather than only from the start of the next iteration. We applied this method to Scenario A as a supplementary analysis to explore its potential benefits. A description of this approach, with accompanying pseudocode, is provided in Supplementary Information A.3.

### Simulation of the Markov decision processes

We created a simulator to represent each scenario. The simulators have two purposes: firstly, to fit parameters for heuristic replenishment policies using simulation optimization and, secondly, to evaluate the performance of the policies produced by value iteration and simulation optimization based on the return and three key performance indicators (KPIs): service level, wastage and holding. The service level is the percentage of demand that was met over a simulated rollout, wastage is the proportion of units received that expired over a simulated rollout and holding is the mean number of units in stock at the end of each day during a simulated rollout.

Each simulator is constructed as a reinforcement learning environment written using the Python library gymnax (Lange, 2022b), which is based on JAX. This provides a standard interface for working with MDPs, while enabling many simulations to be run in parallel on a GPU using vmap. For a single policy, we can use vmap to implement our simulation rollout over multiple random seeds. We can simultaneously evaluate multiple sets of parameters for the same policy on a shared set of random seeds by nesting vmapped functions. We note that it would be straightforward to use reinforcement learning software libraries to learn policies for these scenarios using these environments.

We selected different heuristic policies for the different scenarios from the literature, considering which (if any) heuristic was used in the original study and the structure of each problem. We describe the heuristic policy used for each scenario in the corresponding section below. All of the heuristic policies use one or both of an order-up-to level parameter S and reorder point parameter s. The order quantity is the difference between the current stock on hand and in transit (potentially subject to some modification, as in Scenario B) and the order-up-to level S. If there were no stock on hand or in transit the heuristic policy would order S units, and therefore S corresponds to the largest order that would be placed following the heuristic policy. If the heuristic policy also has a reorder point parameter s then an order is only placed when the current stock on hand and in transit is less than or equal to the reorder point s (Snyder & Shen, 2019).

We used the Python library Optuna (Akiba et al., 2019) to suggest parameters for the heuristic policies. For heuristic policies with a single parameter, we evaluated all feasible values simultaneously in parallel using Optuna’s grid sampler. When there was more than one parameter we instead used a genetic algorithm, Optuna’s Nondominated Sorting Genetic Algorithm II (NSGA-II) (Deb et al., 2002) sampler, to search the parameter space. For each suggested set of parameters, we ran 4000 rollouts, each 365 days long following a warm-up period of 100 days. When using the grid search sampler, we took as the best parameter value the one with the highest mean return after the single parallel run. When using the NSGA-II sampler we ran 50 sets of parameters in parallel, representing a single generation for the genetic algorithm, and ranked them based on the mean return. We terminated the NSGA-II search procedure when the best combination of parameters had not changed for five generations, or when 100 generations had been completed. NSGA-II is designed for multi-ohjective optimization and therefore candidate solutions are selected to generate offspring and/or be carried forward to the next generation based on whether they dominate other candidate solutions and occupy less crowded areas of the solution space to maintain diversity. Despite being designed for multi-objective problems, in preliminary experiments we found NSGA-II to be effective compared to alternatives provided by Optuna because it was able to quickly suggest the next batch of candidate solutions for parallel evaluation.

For each scenario we compare the performance of the value iteration policy and best heuristic policy identified using simulation optimization on 10,000 additional simulated rollouts, each 365 days long following a warm-up period of 100 days. We report the mean and standard deviation of the return, the service level, wastage and stock holding over these rollouts. For each rollout, the return is the discounted sum of rewards from the end of the warm-up period until the end of the simulation. The components of the reward function for Scenarios A, B and C are summarised in Table 2 and the reward functions are set out in Supplementary Information A.1, B.1 and C.1 respectively. The standard deviation of the return and the KPIs shows the effect of the stochasticity (due to random demand, random willingness to accept substitution and/or random useful life on arrival) in each scenario.

### Reproducibility

There are two key reproducibility considerations for this work: firstly, accurately implementing the scenarios described in previous studies and, secondly, ensuring that others are able to reproduce our own experiments.

We compared outputs from our value iteration and simulation optimization methods to outputs from the original studies, and these checks are included as automated tests in our publicly available GitHub repository. De Moor et al. (2022) made their code available on GitHub and fully specified the optimal and heuristic policies for two experiments in their paper which we used to test our implementation of Scenario A. For Scenario B, we compared the best parameters for heuristic policies and mean daily reward values to those reported in Hendrix et al. (2019), and performed additional comparisons to the output of a MATLAB implementation of their value iteration method that the authors kindly made available to us. Mirjalili (2022) plotted value iteration policies for a subset of his experiments, and he kindly provided us with the underlying data for those plots so that we could confirm the policies from our implementation of Scenario C matched those he had reported.

Our code is available on GitHub, and is based on open-source software libraries. Our GitHub repository includes a Google Colab notebook that can be used to reproduce our experiments using a free, cloud-based GPU, avoiding local hardware requirements or configuration challenges.

### Hardware

All experiments were conducted on a desktop computer running Ubuntu 20.04 LTS via Windows Subsystem for Linux on Windows 11 with an AMD Ryzen 9 5900X processor, 64GB RAM, and an Nvidia GeForce RTX 3060 GPU. The Nvidia GeForce RTX 3060 is a consumer-grade GPU that, at the time of writing in December 2024, can be purchased for less than £300 in the United Kingdom (Ebuyer, 2024).

To demonstrate the potential benefits of more powerful data-centre grade GPU devices, and how our approach can be easily scaled to utilise multiple GPUs, we additionally ran value iteration for one large problem case of Scenario B using one, two or four Nvidia A100 40GB GPUs.

## Scenario A: lead time may be greater than one period

### Problem description

De Moor et al. (2022) described a single-product, single-echelon, periodic review perishable inventory replenishment problem and investigated whether using heuristic replenishment policies to shape the reward function can improve the performance of reinforcement learning methods.

At the start of each day *t* the agent observes the state $S_t$, the current inventory in stock (split by remaining useful life) and in transit (split by period ordered), and places a replenishment order $A_t \in \{0, 1,\ldots , A_{\max }\}$. Demand for day *t*, $D_t$, is sampled from a truncated gamma distribution and rounded to the nearest integer. Demand is filled from available stock following either a first-in first-out (FIFO) or last-in first-out (LIFO) issuing policy. At the end of the day, the state is updated to reflect the ageing of stock and the reward, $R_{t+1}$, is calculated. The reward function comprises four components: a holding cost per unit in stock at the end of the period ($C_h$), a variable ordering cost per unit ($C_v$), a shortage cost per unit of unmet demand ($C_s$) and a wastage cost per unit that perishes at the end of the period ($C_w$). The order placed on day $t-(L-1)$ is received immediately prior to the start of day $t+1$, and is included in the stock element of the state $S_{t+1}$.

The stochastic element in the transition is the daily demand *D*, $\mathbb {\Omega } = \{0, 1, \ldots , \infty \}$, in the problem described by De Moor et al. (2022). The state transition and the reward are deterministic given a state-action pair and the realisation of the daily demand. Daily demand is modelled by a gamma distribution with mean $\mu$ and coefficient of variation $\frac{\mu }{\sigma }$. We truncated the demand distribution at $D_{\max } \gg \mu +5\sigma$, such that $\mathbb {\Omega } = \{0, 1,\ldots , D_{\max }\}$, for the purposes of implementation.

The initial value function $V_0(s)$ was initialised at zero for every state. De Moor et al. (2022) did not specify a particular convergence test for their value iteration experiments. The problem is not periodic and includes a discount factor, and we therefore used a standard convergence test for the value function (Sutton & Barto, 2018) as set out in Supplementary Information A.1.

De Moor et al. (2022) considered products with a maximum useful life *m* of two, three, four or five periods, and evaluated eight different experimental settings for each value of *m*. For a product with $m=2$, they found the optimal policy using value iteration, and used this as a benchmark for their deep reinforcement learning policies. For larger values of *m*, they instead used a heuristic policy as the benchmark on grounds of computational feasibility. The experiments for each value of *m* evaluate different combinations of lead time *L*, wastage cost $C_w$, and issuing policy. We demonstrate that, using JAX and a consumer-grade GPU, it is feasible to obtain the optimal policy for all of the experimental settings, up to and including a maximum useful life *m* of five periods, and report the wall time required to run value iteration for each experiment.

We compare the policy from value iteration with a standard base-stock policy, parameterised by order-up-to level $\texttt {S}$, such that the order quantity on day *t*, given total current stock (on hand and in transit) $I_t$ is:

7 $$\begin{aligned} A_t = \left[ \texttt {S} - I_t\right] ^+ \end{aligned}$$

We evaluated the mean return for each value of $\texttt {S} \in \{0,\ldots , A_{\max }\}$ using the Optuna grid sampler. We compare the base-stock policy that achieves the highest mean return, characterised by parameter S$_{\text {best}}$, to the value iteration policy.

See Supplementary Information A.1 for additional information about Scenario A.

### Results

In Table 3 we present the wall time (WT) in seconds required to run value iteration and simulation optimization for each experimental setting. We also present the mean and standard deviation of the return obtained when using value iteration and best heuristic policies on 10,000 simulated rollouts, each 365 days long following a warm-up period of 100 days.

The wall times reported in Table 3 show that, using our approach, the largest cases, with $m=5$ and $L=2$, can be solved using value iteration in under 20 min. The running time for simulation optimization is approximately constant at 2 s over the different problem sizes. This is consistent with the fact that the parameter space for the heuristic base-stock policy is the same for each experimental setting, because the maximum order quantity $A_{\max }$ does not change.

As we would expect, we observe higher mean returns under a FIFO issuing policy than under a LIFO issuing policy and as *m* increases due to lower wastage. The optimality gap is consistently higher for experiments with a longer lead time, suggesting that the age profile of the stock is more important when lead times are longer.

See Supplementary Information A.2 for the best parameters for the heuristic policy and KPIs for each experiment. In the cases with the largest optimality gaps, where $m=L=2$, the improvement is driven by an improved service level at similar levels of stock holding.

We repeated these experiments using asynchronous value iteration and found that it was particularly efficient for the problem settings with the largest number of states, recucing the wall time by up to 39% compared to the results reported in Table 3. The methods and results for asynchronous value iteration are reported in Supplementary Information A.3.

**Table 3:** Our results on Scenario A for all of the experimental settings from De Moor et al. (2022). The longest wall times, for value iteration when $m=5$ and $L=2$, are approximately 20 min. Value iteration was considered intractable for experiments where $m > 2$ in the original study

								Value iteration		Simulation optimization		
*m*	Exp	*L*	$C_w$	Issuing policy	$|\mathbb {S}|$	$|\mathbb {A}|$	$|\mathbb {\Omega }|$	WT (s)	Return	WT (s)	Return	Optimality gap (%)
2	1	1	7	LIFO	121	11	101	5	$-1553 \pm 61$	2	$-1565 \pm 62$	0.80
	2	1	7	FIFO	121	11	101	4	$-1457 \pm 59$	2	$-1474 \pm 56$	1.20
	3	1	10	LIFO	121	11	101	5	$-1571 \pm 61$	2	$-1581 \pm 62$	0.64
	4	1	10	FIFO	121	11	101	5	$-1463 \pm 60$	2	$-1485 \pm 61$	1.46
	5	2	7	LIFO	1331	11	101	5	$-1551 \pm 62$	2	$-1590 \pm 64$	2.49
	6	2	7	FIFO	1331	11	101	5	$-1461 \pm 58$	2	$-1495 \pm 60$	2.31
	7	2	10	LIFO	1331	11	101	6	$-1569 \pm 61$	2	$-1606 \pm 64$	2.35
	8	2	10	FIFO	1331	11	101	5	$-1469 \pm 59$	2	$-1504 \pm 60$	2.41
3	1	1	7	LIFO	1331	11	101	5	$-1490 \pm 58$	2	$-1500 \pm 59$	0.71
	2	1	7	FIFO	1331	11	101	5	$-1424 \pm 56$	2	$-1435 \pm 52$	0.74
	3	1	10	LIFO	1331	11	101	5	$-1498 \pm 61$	2	$-1512 \pm 58$	0.90
	4	1	10	FIFO	1331	11	101	5	$-1425 \pm 55$	2	$-1436 \pm 52$	0.82
	5	2	7	LIFO	14,641	11	101	13	$-1513 \pm 61$	2	$-1533 \pm 61$	1.32
	6	2	7	FIFO	14,641	11	101	13	$-1435 \pm 56$	2	$-1456 \pm 58$	1.42
	7	2	10	LIFO	14,641	11	101	13	$-1526 \pm 60$	2	$-1544 \pm 61$	1.16
	8	2	10	FIFO	14,641	11	101	13	$-1437 \pm 56$	2	$-1457 \pm 58$	1.42
4	1	1	7	LIFO	14,641	11	101	14	$-1459 \pm 56$	2	$-1476 \pm 54$	1.15
	2	1	7	FIFO	14,641	11	101	14	$-1422 \pm 56$	2	$-1430 \pm 52$	0.54
	3	1	10	LIFO	14,641	11	101	14	$-1465 \pm 56$	2	$-1481 \pm 60$	1.08
	4	1	10	FIFO	14,641	11	101	14	$-1422 \pm 56$	2	$-1430 \pm 52$	0.54
	5	2	7	LIFO	161,051	11	101	111	$-1480 \pm 59$	2	$-1496 \pm 59$	1.07
	6	2	7	FIFO	161,051	11	101	110	$-1432 \pm 55$	2	$-1453 \pm 58$	1.44
	7	2	10	LIFO	161,051	11	101	110	$-1489 \pm 59$	2	$-1505 \pm 58$	1.07
	8	2	10	FIFO	161,051	11	101	109	$-1432 \pm 55$	2	$-1453 \pm 58$	1.44
5	1	1	7	LIFO	161,051	11	101	114	$-1443 \pm 55$	2	$-1454 \pm 55$	0.73
	2	1	7	FIFO	161,051	11	101	113	$-1422 \pm 56$	2	$-1430 \pm 52$	0.54
	3	1	10	LIFO	161,051	11	101	114	$-1446 \pm 56$	2	$-1460 \pm 55$	0.94
	4	1	10	FIFO	161,051	11	101	114	$-1422 \pm 56$	2	$-1430 \pm 52$	0.54
	5	2	7	LIFO	1,771,561	11	101	1191	$-1463 \pm 58$	2	$-1480 \pm 60$	1.22
	6	2	7	FIFO	1,771,561	11	101	1185	$-1432 \pm 55$	2	$-1453 \pm 58$	1.44
	7	2	10	LIFO	1,771,561	11	101	1188	$-1467 \pm 58$	2	$-1484 \pm 59$	1.15
	8	2	10	FIFO	1,771,561	11	101	1190	$-1432 \pm 55$	2	$-1453 \pm 58$	1.44

*m*: Maximum useful life, *L*: lead time, $C_w$: wastage cost per unit, $|\mathbb {S}|$: number of possible states, $|\mathbb {A}|$: number of possible actions, $|\mathbb {\Omega }|$: number of possible realisations of stochastic elements in a transition, WT: wall time

## Scenario B: substitution between two perishable products

### Problem description

Hendrix et al. (2019) applied value iteration and simulation optimization to fit replenishment policies for two perishable inventory problems: a single-product scenario that is similar to Scenario A and a scenario with two products and the potential for substitution which we consider here as Scenario B. In Scenario B we manage two perishable products, product A and product B, with the same fixed, known useful life *m*. Some customers who want product B are willing to accept product A instead if product B is out of stock. The lead time $L = 1$ and therefore there is no in-transit component to the state.

At the start of each day *t*, the agent observes state $S_t$, the current inventory of each product in stock split by remaining useful life, and places a replenishment order. The action consists of two elements, one order for each product: $A_t = [A^a_t, A^b_t]$ where $A^a_t \in \{0, 1,\ldots , A^a_{\max }\}$ and $A^b_t \in \{0, 1,\ldots , A^b_{\max }\}$. Demand for day *t* is sampled from independent Poisson distributions for each product, parameterised respectively by mean demand $\mu ^a$ and $\mu ^b$, and is initially filled for each product independently using a FIFO issuing policy. Some customers with unmet demand for product B may be willing to accept product A instead. The substitution demand is sampled from a binomial distribution, with a probability of accepting substitution $\rho$ and a number of trials equal to the unmet demand for product B. After demand for product A has been filled as far as possible, demand for product B willing to accept product A is filled by any remaining units of product A using a FIFO issuing policy. At the end of the day, the state is updated to reflect the ageing of stock, and the reward, $R_{t+1}$ is calculated. The reward function comprises revenue per unit sold ($C_r^a, C_r^b$) and variable order cost ($C_v^a, C_v^b$) for each product. The order placed on day *t* is received immediately prior to the start of day $t+1$ and is included in the stock element of state $S_{t+1}$.

The daily demand and willingness to accept substitution are both stochastic. We capture the effect of both by considering the stochastic element in the transition to be the number of units issued for each product type: $H^a$ and $H^b$. The state transition and the reward are deterministic given a state-action pair and the number of units issued of product A and of product B. The set of possible realisations of the stochastic elements is:

8 $$\begin{aligned} \begin{array}{ll} \mathbb {\Omega } = \{(h^a, h^b)\}\, & \qquad \qquad h^a \in \{0, 1,\ldots , H^a_{\max }=mA^a_{\max }\}\\ & \qquad \qquad h^b \in \{0, 1, \ldots , H^b_{\max }=mA^b_{\max }\} \end{array} \end{aligned}$$

The initial value function, $V_0(s)$, is set to the expected sales revenue for state *s* with $I^a$ units of product A and $I^b$ units of product B in stock. This is an infinite horizon problem with no discount factor, and therefore we used the convergence test specified in Hendrix et al. (2019), which stops value iteration when the value of each state is changing by approximately the same amount on each iteration, indicating a stable estimate of the optimal policy.

Hendrix et al. (2019) considered products with a maximum useful life *m* of two and three periods and evaluated two experimental settings for each value of *m*. For experiments 1 and 2 with $m=3$, where the maximum order quantities were set based on the newsvendor model, they reported that it was not possible to complete value iteration within 1 week. They therefore repeated the two experiments for $m=3$ with lower values of $A^a_{\max }$ and $A^b_{\max }$. With this adjustment, one of the cases could be completed within 80 h, while the other could still not be solved within a week. We demonstrate that using JAX and a consumer-grade GPU it is feasible to obtain the optimal policy for all of these settings and report the wall time required to run value iteration for each experiment. Additionally, to investigate how our method can benefit from more powerful GPUs, and how it scales to multiple GPUs, we report the wall times for running the largest problem on one, two and four Nvidia A100 40GB GPUs.

Separately, we consider the experimental settings used by Ortega et al. (2019) to evaluate their GPU-accelerated method, in which value iteration was always run for 100 iterations instead of to convergence.

In each case, we compare the policy from value iteration with the modified base-stock policy used by Hendrix et al. (2019), based on the work of Haijema and Minner (2019), which has an order-up-to level parameter for each product: $\texttt {S}^a$ and $\texttt {S}^b$. The order quantity for each product is determined considering only the on hand inventory of that product and includes an adjustment for expected waste. The order quantity on day *t*, given total stock on hand $I^a_t$ and $I^a_t$ and stock that expires at the end of the current period $X^a_{1,t}$ and $X^b_{1,t}$, is:

9 $$\begin{aligned} A_t = \left[ A^a_t, A^b_t \right] = \biggl [\Bigl [\texttt {S}^a - I^a_t + \bigl [X^a_{1,t} - \mu ^a\bigr ]^+\Bigr ]^+, \Bigl [\texttt {S}^b - I^b_t + \bigl [X^b_{1,t} - \mu ^b\bigr ]^+\Bigr ]^+\biggr ] \end{aligned}$$

There are two parameters, and we used Optuna’s NSGA-II sampler to search the parameter space $\texttt {S}^a \in \{0, 1, \ldots , \texttt {S}^a_{\max }=2A^a_{\max }\}$ and $\texttt {S}^b \in \{0, 1, \ldots , \texttt {S}^b_{\max }=2 A^b_{\max }\}$. We considered values of the order-up-to level up to twice the maximum order quantity used for value iteration because Hendrix et al. (2019) reported some best values of S that were higher than the values of $A_{\max }$ specified for value iteration. We compare the modified base-stock policy that achieved the highest mean return to the value iteration policy.

See Supplementary Information B.1 for additional information about Scenario B.

### Results

We present results for the experimental settings for the two product scenario from Hendrix et al. (2019) in Table 4. The wall times in Table 4 show that, using our method, value iteration can be used to find the optimal policy for all four settings of the two product scenario with $m=3$ in under 3.2 h. Hendrix et al. (2019) reported that, for $m=3$, value iteration did not converge within a week for experiments 1, 2 and 3 using a MATLAB implementation and experiment 4 converged in 80 h. Our implementation of experiment 4, running on a consumer-grade GPU, converges in just over 2 min: more than 2000${\times }$ faster.

We present results for the four experimental settings, P1 to P4, from Ortega et al. (2019) in Table B3 in Supplementary Information B. The wall times for our approach are at least six times faster than those reported by Ortega et al. (2019) for all four settings. We cannot conclude on the relative performance of our method and the GPU-accelerated method from Ortega et al. (2019) without running both implementations on the same hardware and accounting for the difference between up-front and JIT compilation. However, the results suggest that our method is at least competitive with a custom CUDA implementation of value iteration for the two product case while certainly requiring less specialist knowledge of GPU programming.

Simulation optimization scales well to larger problems, with wall times less than 1 min for all of the experimental settings. The optimality gap is never greater than 1%, and reduces as both mean demand and the maximum useful life increase. This suggests that there is a limited advantage to making ordering decisions based on the age-profile of the stock of both products, compared to making independent decisions for each product using a simple heuristic policy, under the reward function and substitution process proposed by Hendrix et al. (2019).

**Fig. 1:**
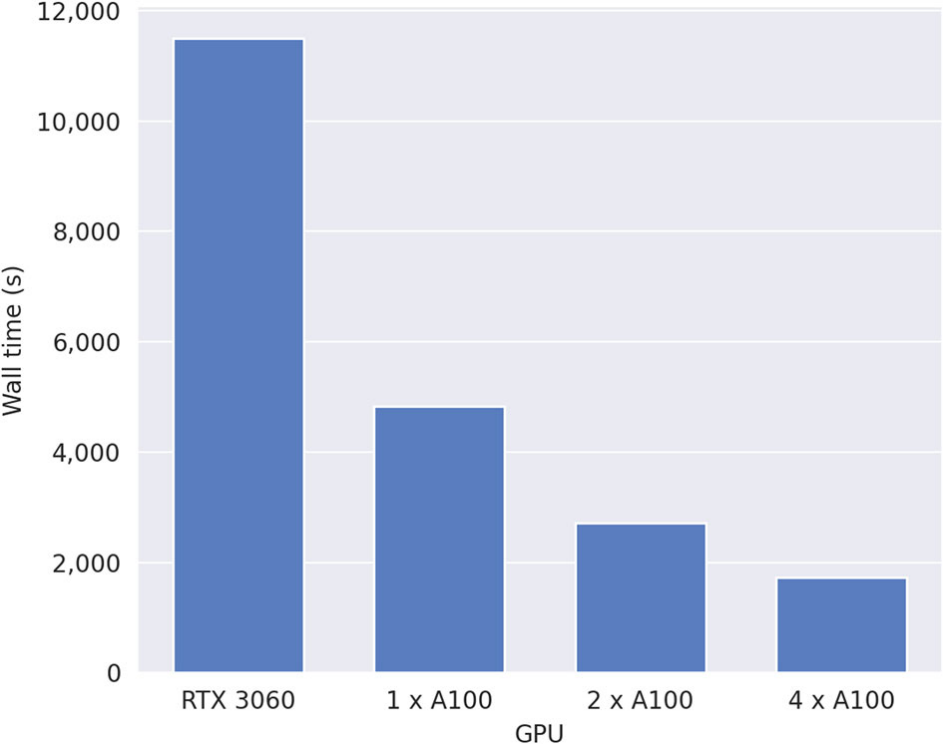
Wall times required to run value iteration for experiment 1 with ${m}{=3}$ for Scenario B using different GPUs. The Nvidia GeForce RTX 3060 is a consumer-grade GPU. The Nvidia A100 40 GB is a data-centre grade GPU. JAX enables our method to be run on multiple identical GPUs without any code changes

Figure 1 illustrates the clear benefits of both more powerful GPUs, and of using multiple GPUs. Using a single Nvidia A100 40GB GPU, experiment 1 when $m=3$ can be run in 4,838 s: 2.4$\times$ faster than the Nvidia RTX 3060 in our local machine. Using two A100 40GB GPUs is 1.8$\times$ faster than one, and using four A100 40GB GPUs is 2.8$\times$ faster than one. The wall time does not decrease linearly with the number of GPUs because not all of the operations are conducted in parallel on GPU—for example at the end of each iteration the updated values need to be aggregated and transferred to each device, and the values are transferred back to CPU in order to save a checkpoint.

See Supplementary Information B.2 for the best parameters for the heuristic policy and the KPIs for each experiment. In the experiments with the largest optimality gaps, when $m=2$, fewer units of product B are ordered under the optimal policy resulting in a lower service level but also less wastage and less holding for product B.

**Table 4:** Our results on Scenario B for all of the experimental settings from Hendrix et al. (2019). The longest wall time, for value iteration on experiment 1 when $m=3$, is approximately 3.2 h. Value iteration could not be completed within a week for experiments 1, 2 and 3, and required 80 h for experiment 4, when $m=3$ in Hendrix et al. (2019)

*m*	Exp	$\mu ^a$	$\mu ^b$	$A^a_{\max }$	$A^b_{\max }$	$|\mathbb {S}|$	$|\mathbb {A}|$	$|\mathbb {\Omega }|$	Value iteration		Simulation optimization		
									WT (s)	Return	WT (s)	Return	Optimality gap (%)
2	1	5	5	10	10	14,641	121	441	5	1644 ± 33	24	1632 ± 34	0.70
	2	7	3	14	6	11,025	105	377	4	1650 ± 33	23	1639 ± 34	0.67
3	1	5	5	15	15	16,777,216	256	2116	11,496	1761 ± 32	33	1,758 ± 32	0.16
	2	7	3	21	9	10,648,000	220	1792	4013	1762 ± 32	44	1759 ± 32	0.18
	3	5	5	13	13	7,529,536	196	1600	3058	1761 ± 32	32	1758 ± 32	0.16
	4	7	3	20	4	1,157,625	105	793	134	1762 ± 32	43	1759 ± 32	0.17

*m*: Maximum useful life, $\mu ^p$: mean demand for product *p*, $A_{\max }^{p}$: maximum order quantity for product *p*, $|\mathbb {S}|$: number of possible states, $|\mathbb {A}|$: number of possible actions, $|\mathbb {\Omega }|$: number of possible realisations of stochastic elements in a transition, WT: wall time

## Scenario C: periodic demand and uncertain useful life on arrival

### Problem description

Mirjalili (2022) described a perishable inventory problem that models the management of platelets in a hospital blood bank. There are two problem features not included in Scenarios A or B. Firstly, the demand is periodic, with an independent demand distribution for each day of the week. Secondly, the remaining useful life of products on arrival is uncertain, and this uncertainty may be exogenous or endogenous. The lead time *L* is assumed to be zero and therefore there is no in transit component to the state.

At the start of each day *t* the agent observes state $S_t$, which specifies the day of the week and the current inventory in stock split by remaining useful life, and places a replenishment order $A_t \in \{0, 1, \ldots , A_{\max }\}$. This order is assumed to arrive instantly. The remaining useful life of the units on arrival is sampled from a multinomial distribution, the parameters of which may depend on the order quantity $A_t$. Demand for day *t*, $D_t$ is sampled from a truncated negative binomial distribution and filled from available stock using an oldest-unit first-out (OUFO) policy. At the end of the day, the state is updated to reflect the ageing of stock and the reward, $R_{t+1}$ is calculated. The reward function comprises four components: a holding cost per unit in stock at the end of the period ($C_h$), a shortage cost per unit of unmet demand ($C_s$), a wastage cost per unit that perishes at the end of the period ($C_w$) and a fixed ordering cost ($C_f$). Unlike Scenario A which also includes a holding cost, the holding cost is charged also on units that expire at the end of the period.

To reduce the number of possible states, we consider a limited case of this problem in which there is a maximum capacity of $A_{\max }$ for stock of each age. If, when an order is received, the sum of units in stock and units received with *k* days of remaining useful life is greater than $A_{\max }$ then we assume the excess units are not accepted at delivery. The stock level with *k* days of remaining useful life is therefore at most $A_{\max }$ when demand is sampled. This constraint is not explicitly stated in the original work, but a capacity constraint is necessary for consistency with the calculation of the total number of states in Mirjalili (2022). There are alternative ways to apply the constraint (e.g. by discarding excess units at the end of each day along with wastage) and these may have different optimal policies.

The stochastic elements in the transition are the daily demand, *D*, and the age profile of the units received to fill the order placed at the start of the day: $\underline{\text {Y}} = \left[ Y_{m}, Y_{m-1}, \ldots , Y_{1}\right]$. The state transition and the reward are deterministic given a state-action pair, the daily demand, and the age profile of the units received. The set of possible realisations of the stochastic elements is:

10 $$\begin{aligned} \begin{array}{ll} \mathbb {\Omega } = \{(d, \underline{\text {y}})\} \, & \quad \qquad d \in \{0, 1, \ldots , D_{\max }\} \\ & \quad \qquad y_i \in \{0, 1, \ldots , A_{\max }\}, \quad \forall i \in \{1, 2, \ldots , m\} \\ & \quad \qquad \sum _{i=1}^{m} y_i \le A_{\max } \end{array} \end{aligned}$$

The initial value function $V_0(s)$ was initialised at zero for every state. Mirjalili (2022) did not specify a particular convergence test for his value iteration experiments. The problem is periodic, with a discount factor, and therefore we use a convergence test based on those described in Su and Deininger (1972) which stops value iteration when the undiscounted change in the value function over a period (in this case, 7 days) is approximately the same for every state. As in Scenario B, when the change in value is the approximately the same for every state there will be no further changes to the best action for every state, and hence, the estimated optimal policy is stable.

Mirjalili (2022) considered products with a maximum useful life of three, five or eight periods and stated that, due to the large state space, value iteration was intractable for this problem when $m \ge 5$. We were able to run value iteration when $m=5$, but not when $m=8$. For each value of *m*, Mirjalili (2022) investigated five different settings for the distribution of useful life upon arrival: one where the uncertainty was exogenous (independent of order quantity), and four where the uncertainty was endogenous (dependent on order quantity). For each of these five settings, he evaluated six combinations of $C_f$ and $C_w$. Our objective was to demonstrate the feasibility of our approach and therefore, given the large number of experiments and long wall times when $m=5$, we ran two experiments for each value of *m*: one where the uncertainty in useful life on arrival was exogenous and one where it was endogenous. For $m=5$, we selected the settings from Mirjalili (2022) that are based on real, observed data from a network of hospitals in Ontario, Canada instead of the additional settings created for sensitivity analysis. We report the wall time required to run value iteration for each experiment.

We compare the policy from value iteration with an $(\texttt {s}, \texttt {S})$ policy. Mirjalili (2022) did not fit heuristic policies, but suggested (s, S) as an example of a suitable heuristic policy for future work: the addition of a fixed ordering cost to the reward function means that it may be beneficial to include the reorder point parameter $\texttt {s}$ to avoid uneconomically small orders. We fit one pair of s and S for each day of the week, a total of 14 parameters. The order quantity on day *t*, given that the day of the week is $\tau$ and the total current stock on hand is $I_t$ is:

11 $$\begin{aligned} A_t = {\left\{ \begin{array}{ll} \left[ \texttt {S}^{\tau } - I_t\right] ^+ & \text { if }I_t \le \texttt {s}^{\tau } \\ 0 & \text { if }I_t > \texttt {s}^{\tau } \end{array}\right. } \end{aligned}$$

where $(\texttt {s}^{\tau }, \texttt {S}^{\tau })$ is the pair of parameters for day of the week $\tau$.

We used Optuna’s NSGA-II sampler to search for combinations of $\texttt {s}^{\tau } \in \{0, 1, \ldots , \texttt {s}_{\max } = A_{\max }\}$ and $\texttt {S}^{\tau } \in \{0, 1, \ldots , \texttt {S}_{\max } = A_{\max }\} \; \forall \tau \in \{0, 1,.., 6\}$. This heuristic policy has a hard constraint that $\texttt {s}^{\tau } < \texttt {S}^{\tau } \; \forall \tau \in \{0, 1,.., 6\}$. Optuna does not support using hard constraints to restrict the search space, so we enforced the constraint by only allowing a non-zero order to be placed if the constraint was met. We compare the heuristic policy that achieved the highest mean return to the value iteration policy.

See Supplementary Information C.1 for additional information about Scenario C. This scenario was taken from Chapter 6 of Mirjalili et al. (2022), but a preprint based on that chapter has now been released (Abouee-Mehrizi et al., 2023).

### Results

In Table 5 we present the results for the experimental settings from Mirjalili (2022) that we have selected, two for each value of *m*. Using our method, it is possible to find the optimal policy using value iteration for $m=3$ and $m=5$ while accounting for uncertainty in useful life on arrival. The experiments where $m=5$ represent a real world problem: Mirjalili (2022) fit the parameters for the demand distribution and distribution of useful life on arrival to observed data from a network of hospitals in Ontario, Canada. This is an important application of our value iteration method, demonstrating that it can be used to find optimal policies for problems of a realistic size. The alternative experimental settings evaluated by Mirjalili (2022) but not repeated here have the same numbers of states, actions and possible random outcomes and therefore we would expect the wall times to be of a similar order as corresponding experiments reported in Table 5.

We were unable to complete value iteration when $m=8$. This problem has over 12.6 billion possible states, even with the restriction that we placed on the maximum stock holding of each age, and over 65 million possible random outcomes. It is not feasible to store the state array in the memory of our local machine, let alone run value iteration. However, we were able to fit a heuristic policy using simulation optimization in less than 20 min.

The simulation optimization experiments for this scenario take longer than those of the other scenarios, between 5 and 20 min. This is due to the large number of possible combinations of parameters, because our heuristic policy require seven pairs of parameters $\left( \texttt {s},\texttt {S}\right)$, one for each weekday. The size of the search space for each experiment is $(A_{\max } + 1)^{14} = 3.2 \times 10^{18}$, compared to only 11 possible parameters for the base-stock policy used for Scenario A and fewer than 1000 possible combinations of parameters for even the largest scenarios from Scenario B. The heuristic policies perform well, with a maximum optimality gap of 1.22%.

See Supplementary Information C.2s for the best parameters for the heuristic policy and KPIs for each experiment. For the experiments with the highest optimality gaps, when $m=5$, the optimal policy achieves better performance when the distribution of remaining useful life on arrival is exogenous due to a higher service level at the cost of more wastage and higher stockholding. Conversely, when the distribution of remaining useful life on arrival is endogenous, the optimal policy achieves a lower service level, but with lower wastage and stockholding.

**Table 5:** Our results on Scenario C for a subset of the experimental settings from Mirjalili (2022): two examples for each value of *m*. The longest wall time, for value iteration on experiment 1 when $m=5$, is approximately 49.5 h. Value iteration was considered intractable for the experiments where $m \ge 5$ in the original study. We were able to use value iteration when $m=5$, but not when $m=8$

*m*	Exp	Uncertainty in useful life	$|\mathbb {S}|$	$|\mathbb {A}|$	$|\mathbb {\Omega }|$	Value iteration		Simulation optimization		
						WT (s)	Mean return	WT (s)	Mean return	Optimality gap (%)
3	1	Exogenous	3087	21	37,191	15	$-410 \pm 62$	507	$-411 \pm 63$	0.26
	2	Endogenous	3087	21	37,191	17	$-349 \pm 53$	305	$-352 \pm 55$	1.04
5	1	Exogenous	1,361,367	21	1,115,730	178,078	$-312 \pm 46$	514	$-313 \pm 50$	0.34
	2	Endogenous	1,361,367	21	1,115,730	178,023	$-312 \pm 47$	393	$-315 \pm 46$	1.22
8	1	Exogenous	12,607,619,787	21	65,270,205	–	–	618	$-293 \pm 42$	–
	2	Endogenous	12,607,619,787	21	65,270,205	–	–	972	$-297 \pm 43$	–

*m*: Maximum useful life, $|\mathbb {S}|$: number of possible states, $|\mathbb {A}|$: number of possible actions, $|\mathbb {\Omega }|$: number of possible realisations of stochastic elements in a transition, WT: wall time

**Fig. 2:**
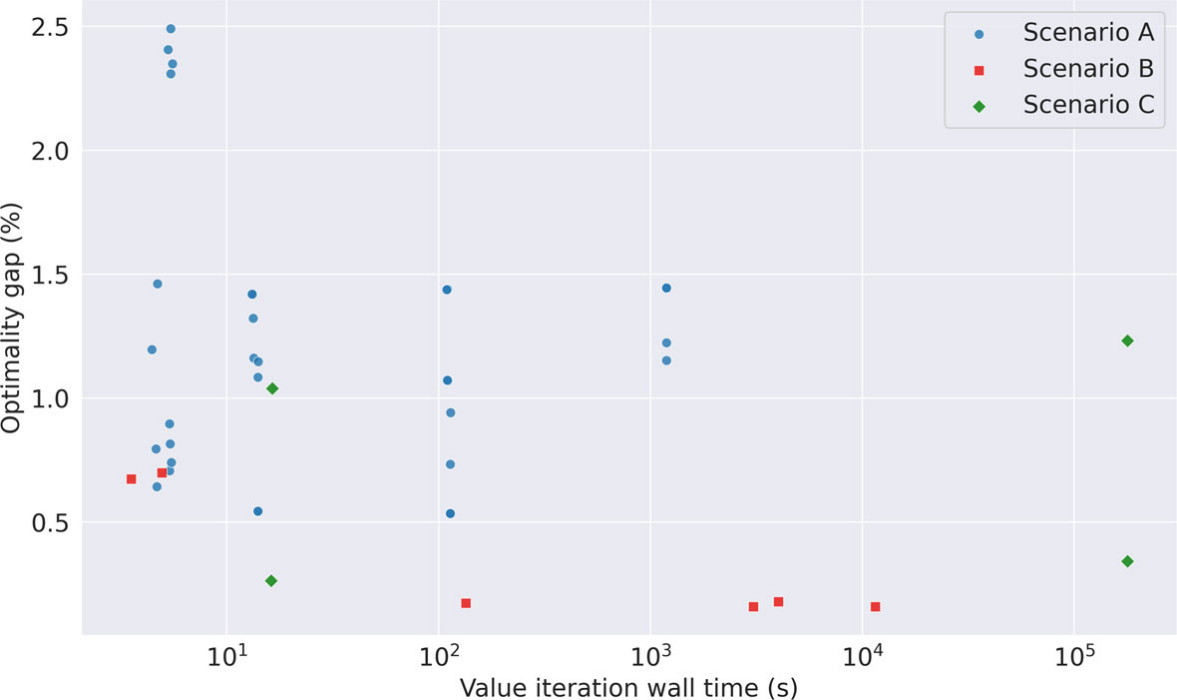
The optimality gap between the heuristic policy that achieved the highest mean return and the value iteration policy plotted against the wall time required to run value iteration for the experiments from Scenarios A, B and C

In Fig. 2 we draw together the results from Scenario C with those from the preceding scenarios, and plot the optimality gap between the heuristic policy that achieved the highest mean return and the value iteration policy against the wall time required for value iteration.

## Discussion

We have found JAX to provide an effective way to expand the scale of perishable inventory problems for which value iteration is tractable, using only consumer-grade hardware and without the need for expertise in GPU programming. By developing a faster, parallel implementation of value iteration we have been able to see further: obtaining the optimal policies for large problems that were previously considered infeasible or impractical. A key benefit of finding these policies is being able to properly quantify the performance of heuristic and approximate policies, in terms of the optimality gap and difference in KPIs, on larger problem instances. Additionally, using these metrics, it is possible to investigate how the relative performance of heuristic and approximate policies scales with properties that influence the problem size. This may support the development of new heuristics, and determining the utility of reinforcement learning and other approximate methods.

Expanding the range of problems to which value iteration can be applied is not just a matter of considering settings with greater demand, more products, or products with a longer useful life. It also enables us to incorporate real-world complexity that might otherwise be neglected due to its effect on the computational tractability such as substitution and endogenous uncertainty in the useful life of products on arrival. An “optimal” policy fit using value iteration is optimal for the situation as modelled, but may not perform well in practice if the model neglects challenging aspects of the real problem. For example, Mirjalili (2022) reported large optimality gaps, with an average of 51%, when policies obtained under the assumption that all stock arrived fresh were applied to a scenario with endogenous uncertainty in useful life. One avenue for future work is to consider scenarios that combine the more challenging elements: a multi-period lead time, substitution between multiple products, and uncertainty in the useful life on arrival which may all be relevant to managing blood product inventory in reality.

The optimality gap in our experiments was never larger than 2.5%, and in the experiments from Scenario A and Scenario B the optimality gap decreased as the demand and/or maximum useful life of the product increased. This is encouraging because it suggests that in some circumstances where the problem size remains too large for value iteration there may actually be little to gain by using the optimal policy over one of these heuristic policies. In Scenario C, the optimality gaps increased as the maximum useful life increased, suggesting that the heuristic policies may be less effective on larger problems when the stock is now always fresh on arrival.

One limitation of this work is that we have not directly compared the performance of the GPU-accelerated methods implemented in JAX against our own CPU-based approach. As we discuss in Sect. 2, previous studies have established the performance advantages of a GPU-based approach to value iteration over a CPU-based approach for problems on which both are feasible. Our primary aim was therefore to develop an accessible method for solving problems that had been described in recent studies as impractical or infeasible using CPU-based approaches. Accepting the claims of prior work, the expected outcome of CPU-benchmarking for the largest problems would be to expend a large amount of computational time just to establish some time period within which these problems could not be solved on CPU. For the smaller problem sizes, a remaining challenge would be selecting the most appropriate benchmark: the studies from which the scenarios were drawn used a range of different languages (MATLAB, Python, C) and we would expect the performance under these different languages (and, particularly for Python, the choice of libraries) to vary significantly. We therefore chose to focus on comparing the performance metrics and wall time of value iteration and simulation optimization approaches, and report wall times for realistic use cases (e.g. including JIT compilation and recording checkpoints) on affordable hardware which we believe will be most relevant to researchers considering applying this computational approach on their own problems.

A second limitation is that in our simulation optimization experiments we only used GPUs to run the simulated rollouts. The heuristic search methods for proposing the next sets of candidate parameters are CPU-based. We did not find Optuna’s NSGA-II sampler to be a bottleneck, but during preliminary experiments we found that some alternative methods took longer to propose the next set of candidate parameters than was required to evaluate them on simulated rollouts. In future work, the optimization process suggesting parameters could also be run on GPU similar to the work of Lau and Srinivasan (2016) and recent work using evolutionary strategies on GPUs to search for neural network parameters (Lange, 2022a). The gymnax-based simulators would also be well suited to ranking and selection methods because it would be straightforward to run a small number of rollouts for a large number of possible parameters in parallel and then, at a second stage, run a large number of rollouts for the most competitive parameters in parallel to obtain more accurate estimates of their performance.

One of the main contributions of this work is to demonstrate an accessible way of using GPUs to accelerate value iteration and simulation optimization and therefore solve larger problems that are closer to those faced in reality. On the software side, we implemented our approach using the relatively high-level JAX API and relied on the XLA compiler to efficiently utilise GPU hardware. On the hardware side, we primarily report results on a consumer-grade GPU, and make available a Google Colab notebook so that our experiments can be reproduced at no cost using cloud-based computational resources. However, a significant strength of JAX is support for easily distributing a workload over multiple identical GPU devices using the pmap function transformation and we discuss in Sect. 5 how additional devices can be used to further reduce the wall time and potentially make even larger problems tractable. Modern cloud computing platforms provide on-demand access to data-centre grade GPUs, including the A100 40GB GPU we used to run the scaling experiments in Sect. 5. At the time of writing in December 2024, a single A100 40GB GPU is available on-demand for $3.67 per hour through Google Cloud Platform (Shen, 2024). This may provide a cost-effective way for research teams without access to local high-performance computing resources to investigate problems that are too large for freely available or consumer-grade GPU hardware.

While we focused on standard value iteration, alternative exact (e.g. Howard policy iteration) and approximate (e.g. optimistic policy iteration) algorithms for solving dynamic programming problems which could also be implemented using JAX may offer further efficiency gains (Sargent & Stachurski, 2024). Our supplementary analysis for Scenario A found that the exact method of asynchronous value iteration implemented in JAX was up to 39% faster than standard value iteration on the largest problem settings.

Future hardware development will make value iteration feasible for even larger problems. In addition to future generations of GPUs, one promising direction is field programmable gate arrays (FPGAs): integrated circuits that can be reprogrammed to customise the hardware to implement a specific algorithm, including value iteration (Peri, 2020). Customising the hardware currently requires specialist knowledge but, just as machine learning frameworks and higher-level tools have made GPU programming more accessible, Peri (2020) suggests that FPGA compilers able to translate high level code into customised circuit designs may facilitate wider adoption.

We have focused on perishable inventory management in this study, but our computational approach has much wider applicability. For each scenario we created a custom subclass of our base value iteration runner class and a custom subclass of the gymnax reinforcement learning environment as our simulator, each with methods to implement the scenario-specific logic. This approach could be followed for other problems that can be modelled as an MDP and where finding the optimal policy for larger settings of interest has recently been described as infeasible or impractical (Liu & Papier, 2022; Liu et al., 2019; Voelkel et al., 2020). For other problems, where realistic settings are likely to remain infeasible for the foreseeable future, GPU-accelerated value iteration may be useful for understanding the scaling properties of proposed heuristic policies on a wider range of example problems (Heydar et al., 2022; Lodree et al., 2019). More broadly, we believe that JAX (and other software libraries originally developed to support deep learning including PyTorch and Tensorflow) offers an efficient way for researchers to run large workloads in parallel on relatively affordable GPU hardware which may support research on a range of operational research problems.

## Conclusion

JAX and similar software libraries provide a way for operational researchers to take advantage of the parallel processing capabilities of modern GPUs without needing extensive experience of GPU programming. In this study we have shown how a JAX-based approach can expand the range of perishable inventory management problems for which value iteration is tractable, using only consumer-grade hardware. We also created GPU-accelerated simulators for each scenario, in the form of JAX-based reinforcement learning environments, and demonstrated how these can be used to fit quickly the parameters of heuristic policies by simultaneously evaluating many sets of policy parameters on thousands of simulated rollouts in parallel. By reducing the wall time required to run value iteration and simulation optimization, these methods can support research into even larger problems, both in terms of scale and the incorporation of aspects of reality that increase the computational complexity. The ability to find optimal policies using value iteration may provide a valuable benchmark for the evaluation of new heuristic and approximate methods, helping efforts to make the best use of scarce resources and reduce wastage of perishable inventory. This work is focused on perishable inventory management but we believe that our methods, and the underlying principle of using software developed by the machine learning community to parallelize workloads on GPU, may be applicable to many other problems in operational research and we have made our code publicly available to support future work.

## Supplementary Information

Below is the link to the electronic supplementary material.

Supplementary file 1 (pdf 406 KB)

## Data Availability

All data supporting the findings of this study are available within the paper and its Supplementary Information. The code used to generate the results is available at https://github.com/joefarrington/viso_jax.

## Footnotes

1 This name of this library is usually written in upper case letters but it is not an acronym.
