# Supplementary material for: Going faster to see further: graphics processing unit-accelerated value iteration and simulation for perishable inventory control using JAX
Source: Ann Oper Res. 2025 Mar 24;349(3):1609–38. doi: 10.1007/s10479-025-06551-6 (PMC12350524; doi:10.1007/s10479-025-06551-6)
Supplement: Supplementary file 1 — (pdf 406 KB) [file 10479_2025_6551_MOESM1_ESM.pdf]

Going faster to see further: Graphics processing unit-accelerated value iteration and simulation for perishable inventory control using JAX

Annals of Operations Research

Supplementary Information

Joseph Farrington[a*], Wai Keong Wong[a,b,c], Kezhi Li[a#], Martin Utley[d]

[a]Institute of Health Informatics, University College London, London, UK
[b]NIHR University College London Hospitals Biomedical Research Centre, University College London and University College London Hospitals NHS Foundation Trust, London, UK
[c]Cambridge University Hospitals NHS Foundation Trust, Cambridge, UK
[d]Clinical Operational Research Unit, University College London, London, UK

*Corresponding author: ucabjmf@ucl.ac.uk (ORCID: 0000-0003-4156-3419)
Primary supervisor: ken.li@ucl.ac.uk

A Additional information for Scenario A

A.1 Scenario description

In this section we recast the problem formulated by De Moor et al. (2022) into a consistent notation used for all three of the scenarios.

The state of the system, S_t, comprises two components: the orders in transit $\underline{\mathrm{O}_t}$ and the units in stock $\underline{\mathrm{X}_t}$:

$$\underline{\mathrm{O}_t} = [O_{L-1,t} = A_{t-1}, O_{L-2,t}, ..., O_{1,t}] \tag{A.1}$$
$$\underline{\mathrm{X}_t} = [X_{m,t} = O_{1,t-1}, X_{m-1,t}, ..., X_{1,t}] \tag{A.2}$$

for a total of $(m + L - 1)$ elements, with lead time $L \geq 1$. The total number of possible states is therefore $(A_{\max} + 1)^{m+L-1}$. In the state $S_t = [\underline{\mathrm{O}_t}, \underline{\mathrm{X}_t}]$, the entries are ordered by ascending age: the first element is the order placed on day $t - 1$ and the last element is the stock that will expire at the end of the current day. The total number of units in stock at the start of day t is $X_t = \sum_{i=1}^{m} X_{i,t}$, the total number of units in transit at the start of day t is $O_t = \sum_{i=1}^{L-1} O_{i,t}$. The total number of units in stock or in transit at the start of day t is $I_t = X_t + O_t$. If $L = 1$ there is no in transit component to the state, and the first element of $\underline{\mathrm{X}_t}$ is A_{t-1}. In Table A1 we present the parameter values that are the same for all of the experiments for Scenario A.

	$D_{\max}$	$A_{\max}$	C_v	C_s	C_h	μ	$\frac{\mu}{\sigma}$	γ	ϵ
Value	100	10	3	5	1	4	0.5	0.99	1×10^{-4}

Table A1: Parameter values that are consistent for all of the experiments for Scenario A.

Daily demand, the stochastic element in the transition, is modelled using a truncated gamma distribution. It does not depend on the state or the action. The demand for the product is discrete and the gamma distribution is continuous, so the probability that the daily demand is equal to $d \in \{0, 1, ..., D_{\max}\}$ is:

$$\begin{aligned}\mathrm{Prob}(\Omega = \omega | S = s, A = a) &= P(\Omega = d) \\ &= P(D = d) \\ &= \begin{cases} F(d + \frac{1}{2}; \mu, \frac{\mu}{\sigma}) - F(d - \frac{1}{2}; \mu, \frac{\mu}{\sigma}), & \text{if } d \in \{0, 1, ..., D_{\max} - 1\} \\ 1 - F(D_{\max} - \frac{1}{2}; \mu, \frac{\mu}{\sigma}), & \text{if } d = D_{\max} \end{cases}\end{aligned} \tag{A.3}$$

where $F(x; \mu, \frac{\mu}{\sigma})$ is the cumulative distribution function of the gamma distribution parameterised by mean μ and coefficient of variation $\frac{\mu}{\sigma}$, and $F(x; \mu, \frac{\mu}{\sigma}) = 0$ when $x \leq 0$.

The reward function comprises four components: a holding cost per unit in stock at the end of the period (C_h), a variable ordering cost per unit (C_v), a shortage cost per unit of unmet demand (C_s) and a wastage cost per unit that perishes at the end of the period (C_w). The single-step reward after taking action A_t in state S_t with $\Omega_t = (D_t)$ is:

$$R_{t+1} = -C_v A_t - C_h \left[X_t - D_t - W_t\right]^+ - C_s \left[D_t - X_t\right]^+ - C_w W_t \tag{A.4}$$

where W_t is the number of units that expire at the end of period t.

Equations A.5 and A.6 set out how the number of expired units, W_t, is calculated and how the elements of $\underline{\mathrm{X}_t}$ are updated when following a FIFO issuing policy and a LIFO issuing policy, respectively.

$$
\begin{aligned}
W_t &= [X_{1,t} - D_t]^+ \\
X_{j,t+1} &= \left[X_{j+1,t} - \left[D_t - \sum_{k=1}^{j} X_{k,t}\right]^+\right]^+ \quad \forall j \in \{1,2,...,m-1\} \\
X_{m,t+1} &= O_{1,t} = A_{t-L+1}
\end{aligned} \tag{A.5}
$$

$$
\begin{aligned}
W_t &= \left[X_{1,t} - \left[D_t - \sum_{k=2}^{m} X_{k,t}\right]^+\right]^+ \\
X_{j,t+1} &= \left[X_{j+1,t} - \left[D_t - \sum_{k=j+2}^{m} X_{k,t}\right]^+\right]^+ \quad \forall j \in \{1,2,...,m-1\} \\
X_{m,t+1} &= O_{1,t} = A_{t-L+1}
\end{aligned} \tag{A.6}
$$

The scenario is an infinite horizon MDP with a discount factor and no periodicity in the state space. We therefore used a standard convergence test for the value function (Sutton & Barto, 2018), evaluating:

$$
\max_{s \in \mathbb{S}} |V_i(s) - V_{i-1}(s)| < \epsilon \tag{A.7}
$$

after each iteration. The inequality tests for the convergence of the values themselves, and requires more iterations than the convergence tests used for the other scenarios which are testing for convergence of the change in value for each state. The test compares the current estimate of the value function with the estimate from the immediately preceding iteration and does not require previous checkpoints for evaluation. Therefore, to save storage space and writing time, we saved a checkpoint every 100 iterations.

A.2 Additional results

We present additional results for Scenario A in Table A2: the order-up-to level parameter $\mathtt{S}_{\text{best}}$ fit using simulation optimization and the mean and standard deviation of three KPIs calculated over 10,000 evaluation rollouts for each policy.

A.3 Asynchronous value iteration

A.3.1 Methods

We implemented asynchronous value iteration using JAX, extending our implementation of value iteration. The pseudocode for this implementation is set out in Algorithm A.1. The pseudocode explicitly sets out the details for multiple devices and batches because these settings affect the updates. In standard value iteration the same estimate of the value function is used for all the updates in an iteration and therefore the batch size and number of devices may affect the wall time but do not affect the updates or the number of iterations required for convergence. For asynchronous value iteration, the updated value of a state is used to update states in subsequent batches on the same device. The choice of batch size and number of devices affect how much updated information is available, and therefore potentially the number of iterations required for convergence.

We developed a subclass of `ValueIterationRunner`, `AsyncValueIterationRunner`, that implements the logic set out in Algorithm A.1. The `AsyncValueIterationRunner` class for Scenario A inherits methods from both the standard `ValueIterationRunner` for Scenario A

m	Exp	S_{best}	Service level (%) VI	Service level (%) SO	Wastage (%) VI	Wastage (%) SO	Holding (units) VI	Holding (units) SO
2	1	5	61.0 ± 1.4	58.6 ± 1.3	2.4 ± 0.6	2.2 ± 0.6	0.2 ± 0.0	0.2 ± 0.0
	2	7	72.7 ± 1.6	76.6 ± 1.5	0.7 ± 0.4	1.5 ± 0.5	0.5 ± 0.1	0.8 ± 0.1
	3	5	61.0 ± 1.4	58.6 ± 1.3	2.4 ± 0.6	2.2 ± 0.6	0.2 ± 0.0	0.2 ± 0.0
	4	6	71.7 ± 1.6	68.6 ± 1.5	0.7 ± 0.3	0.7 ± 0.3	0.5 ± 0.1	0.5 ± 0.0
	5	7	61.0 ± 1.4	55.4 ± 1.3	2.4 ± 0.6	2.4 ± 0.7	0.2 ± 0.0	0.2 ± 0.0
	6	9	73.5 ± 1.7	69.4 ± 1.5	0.9 ± 0.4	1.1 ± 0.4	0.6 ± 0.1	0.6 ± 0.1
	7	7	61.0 ± 1.4	55.4 ± 1.3	2.4 ± 0.6	2.4 ± 0.7	0.2 ± 0.0	0.2 ± 0.0
	8	9	72.3 ± 1.6	69.4 ± 1.5	0.8 ± 0.4	1.1 ± 0.4	0.6 ± 0.1	0.6 ± 0.1
3	1	6	69.5 ± 1.5	68.3 ± 1.4	1.3 ± 0.4	1.4 ± 0.4	0.5 ± 0.1	0.5 ± 0.0
	2	8	79.3 ± 1.5	83.3 ± 1.4	0.1 ± 0.1	0.2 ± 0.2	0.9 ± 0.1	1.3 ± 0.1
	3	6	65.2 ± 1.4	68.3 ± 1.4	0.7 ± 0.3	1.4 ± 0.4	0.4 ± 0.0	0.5 ± 0.0
	4	8	79.3 ± 1.5	83.3 ± 1.4	0.1 ± 0.1	0.2 ± 0.2	0.9 ± 0.1	1.3 ± 0.1
	5	8	65.6 ± 1.6	62.6 ± 1.4	1.7 ± 0.5	1.4 ± 0.5	0.3 ± 0.0	0.4 ± 0.0
	6	10	78.1 ± 1.6	75.5 ± 1.5	0.1 ± 0.1	0.1 ± 0.1	0.9 ± 0.1	0.9 ± 0.1
	7	8	65.6 ± 1.6	62.6 ± 1.4	1.7 ± 0.5	1.4 ± 0.5	0.3 ± 0.0	0.4 ± 0.0
	8	10	77.9 ± 1.6	75.5 ± 1.5	0.1 ± 0.1	0.1 ± 0.1	0.9 ± 0.1	0.9 ± 0.1
4	1	7	74.4 ± 1.4	76.4 ± 1.5	0.7 ± 0.3	1.5 ± 0.4	0.7 ± 0.1	0.8 ± 0.1
	2	8	79.3 ± 1.5	83.3 ± 1.4	0.0 ± 0.0	0.0 ± 0.0	0.9 ± 0.1	1.3 ± 0.1
	3	6	73.7 ± 1.4	68.5 ± 1.4	0.6 ± 0.3	0.5 ± 0.3	0.7 ± 0.1	0.5 ± 0.1
	4	8	79.3 ± 1.5	83.3 ± 1.4	0.0 ± 0.0	0.0 ± 0.0	0.9 ± 0.1	1.3 ± 0.1
	5	9	69.5 ± 1.5	69.3 ± 1.5	1.0 ± 0.4	1.0 ± 0.4	0.5 ± 0.1	0.6 ± 0.1
	6	10	78.9 ± 1.7	75.5 ± 1.5	0.0 ± 0.0	0.0 ± 0.0	1.0 ± 0.1	0.9 ± 0.1
	7	9	68.7 ± 1.5	69.3 ± 1.5	0.9 ± 0.4	1.0 ± 0.4	0.5 ± 0.1	0.6 ± 0.1
	8	10	78.9 ± 1.7	75.5 ± 1.5	0.0 ± 0.0	0.0 ± 0.0	1.0 ± 0.1	0.9 ± 0.1
5	1	7	76.3 ± 1.5	76.6 ± 1.5	0.4 ± 0.2	0.7 ± 0.3	0.8 ± 0.1	0.8 ± 0.1
	2	8	79.3 ± 1.5	83.3 ± 1.4	0.0 ± 0.0	0.0 ± 0.0	0.9 ± 0.1	1.3 ± 0.1
	3	7	75.6 ± 1.4	76.6 ± 1.5	0.3 ± 0.2	0.7 ± 0.3	0.8 ± 0.1	0.8 ± 0.1
	4	8	79.3 ± 1.5	83.3 ± 1.4	0.0 ± 0.0	0.0 ± 0.0	0.9 ± 0.1	1.3 ± 0.1
	5	9	71.9 ± 1.6	69.5 ± 1.5	0.6 ± 0.3	0.4 ± 0.3	0.6 ± 0.1	0.6 ± 0.1
	6	10	78.9 ± 1.7	75.5 ± 1.5	0.0 ± 0.0	0.0 ± 0.0	1.0 ± 0.1	0.9 ± 0.1
	7	9	71.5 ± 1.6	69.5 ± 1.5	0.5 ± 0.3	0.4 ± 0.3	0.6 ± 0.1	0.6 ± 0.1
	8	10	78.9 ± 1.7	75.5 ± 1.5	0.0 ± 0.0	0.0 ± 0.0	1.0 ± 0.1	0.9 ± 0.1

Table A2: The best order-up-to level S_{best}, fit using simulation optimization, and KPIs for policies fit using value iteration (VI) and simulation optimization (SO) for all of the experimental settings for Scenario A from De Moor et al. (2022).

(the scenario-specific logic) and from the base `AsyncValueIterationRunner` (the logic for asynchronous updates).

We repeated the experiments performed in Section 4 using asynchronous value iteration. We used the same hardware (including a single GPU device), the same batch sizes as the standard value iteration experiments reported in Table 3, and the same convergence test to support a direct comparison. The convergence tests for Scenarios B and C could not be straightforwardly applied to asynchronous value iteration, and therefore the method was only used to find policies for Scenario A.

A.3.2 Results

In Table A3 we present the percentage difference in the number of iterations required for convergence and the total wall time when using asynchronous value iteration as compared to standard value iteration for the Scenario A experiments. As expected, the policies found were identical in each case.

For the smallest settings, where all states are updated simultaneously in a single batch, asynchronous value iteration performs the same updates as standard value iteration. The number of iterations required therefore does not change. However, there is a small increase in wall time due to the additional overhead required to support asynchronous updates.

For the larger settings, where each iteration involves updating multiple batches of states,

Algorithm A.1 Asynchronous value iteration using pmap and vmap

Initialise array of all states s: $\mathbb{S}$
Initialise array of all actions a: $\mathbb{A}$
Initialise array of all random outcomes ω: Ω
Initialise initial estimate of value function: $V_0(s) \quad \forall s \in \mathbb{S}$
Initialise discount factor: γ
Initialise iteration counter: $i = 0$
Define deterministic transition function which returns next state and reward: $T(s, a, \omega)$
Set number of devices: Z
Set number of batches: B

Perform value iteration
while not converged **do**
  $i \leftarrow i + 1$
  Divide $\mathbb{S}$ into $Z \times B$ batches $\mathbb{S}_{z,b}$, where $z \in \{1, ..., Z\}$ and $b \in \{1, ..., B\}$
  pmap over $z \in \{1, ..., Z\}$
    $V_i^z(s) \leftarrow V_{i-1}(s),\ \forall s \in \mathbb{S}$ ▷ Each device gets a copy of the value function
    for each batch b **on device** z **do**
      vmap over $s \in \mathbb{S}_{z,b}$
        vmap over $a \in \mathbb{A}$
          vmap over $\omega \in \Omega$
            $(s'_\omega, r_\omega) \leftarrow T(s, a, \omega)$
          $Q_i(s, a) \leftarrow \sum_\omega P(\omega|s, a)\, [r_\omega + \gamma V_i^z(s'_\omega)]$
        $V_i^z(s) \leftarrow \max_a Q_i(s, a)$ ▷ Updated values available to subsequent batches on same device
    end for
  $V_i(s) \leftarrow \text{Aggregate}(\{V_i^z(s)\}, \forall z \in \{1, ..., Z\})$ ▷ Merge updated values from all devices
  Test for convergence
end while

Extract the policy, $\pi(s) \approx \pi^*(s)$ ▷ Following approach in Algorithm 1

this additional overhead is more than offset by a reduction in the number of iterations required for convergence. As a result, asynchronous value iteration achieves time savings of up to 39%.

In this supplementary analysis we used the same batch size for both methods to make the comparison straightforward. The batch size affects the convergence speed of asynchronous value iteration and, in future work, it may be beneficial to investigate this impact further. Large batch sizes make more efficient use of the GPU hardware, but smaller batch sizes mean that more states benefit from updated information within an iteration.

m	Exp	L	C_w	Issuing policy	$\lvert\mathbb{S}\rvert$	$\lvert\mathbb{A}\rvert$	$\lvert\Omega\rvert$	**Percentage reduction (increase)**	
								Iterations	Wall time
2	1	1	LIFO	7	121	11	101	0	(5)
	2	1	LIFO	7	121	11	101	0	(8)
	3	1	LIFO	10	121	11	101	0	(3)
	4	1	LIFO	10	121	11	101	0	(5)
	5	2	LIFO	7	1,331	11	101	0	(9)
	6	2	LIFO	7	1,331	11	101	0	(4)
	7	2	LIFO	10	1,331	11	101	0	(10)
	8	2	LIFO	10	1,331	11	101	0	(8)
3	1	1	LIFO	7	1,331	11	101	0	(9)
	2	1	LIFO	7	1,331	11	101	0	(15)
	3	1	LIFO	10	1,331	11	101	0	(8)
	4	1	LIFO	10	1,331	11	101	0	(10)
	5	2	LIFO	7	14,641	11	101	23	15
	6	2	LIFO	7	14,641	11	101	20	12
	7	2	LIFO	10	14,641	11	101	25	14
	8	2	LIFO	10	14,641	11	101	20	10
4	1	1	LIFO	7	14,641	11	101	37	26
	2	1	LIFO	7	14,641	11	101	31	21
	3	1	LIFO	10	14,641	11	101	38	27
	4	1	LIFO	10	14,641	11	101	31	23
	5	2	LIFO	7	161,051	11	101	14	10
	6	2	LIFO	7	161,051	11	101	38	35
	7	2	LIFO	10	161,051	11	101	14	10
	8	2	LIFO	10	161,051	11	101	38	36
5	1	1	LIFO	7	161,051	11	101	41	37
	2	1	LIFO	7	161,051	11	101	32	28
	3	1	LIFO	10	161,051	11	101	41	37
	4	1	LIFO	10	161,051	11	101	32	28
	5	2	LIFO	7	1,771,561	11	101	25	20
	6	2	LIFO	7	1,771,561	11	101	41	37
	7	2	LIFO	10	1,771,561	11	101	24	19
	8	2	LIFO	10	1,771,561	11	101	41	39

Abbreviations: m: maximum useful life, L: lead time, C_w: wastage cost per unit, $\lvert\mathbb{S}\rvert$: number of possible states, $\lvert\mathbb{A}\rvert$: number of possible actions, $\lvert\Omega\rvert$: number of possible realisations of stochastic elements in a transition.

Table A3: Percentage reduction in iterations required for convergence and wall time on Scenario A when using asynchronous value iteration (Algorithm A.1) compared to standard value iteration (Algorithm 1) for all of the experimental settings from De Moor et al. (2022).

B Additional information for Scenario B

B.1 Scenario description

In this section we recast the problem formulated by Hendrix et al. (2019) into a consistent notation used for all three of the scenarios.

The state of the environment, S_t comprises two components, one for each product type. In the combined state $S_t = \left[\underline{\mathrm{X}}_t^a, \underline{\mathrm{X}}_t^b\right]$, the elements in each component are ordered by ascending age:

$$\underline{\mathrm{X}}_t^a = [X_{m,t}^a = A_{t-1}^a, X_{m-1,t}^a, ..., X_{1,t}^a] \tag{B.1}$$
$$\underline{\mathrm{X}}_t^b = [X_{m,t}^b = A_{t-1}^b, X_{m-1,t}^b, ..., X_{1,t}^b] \tag{B.2}$$

for a total number of $2m$ elements. The total number of possible states is therefore $(A_{\max}^a + 1)^m + (A_{\max}^b + 1)^m$. The total number of units in stock at the start of period t is $I_t^a = X_t^a = \sum_{i=1}^m X_{i,t}^a$ for product A and $I_t^b = X_t^b = \sum_{i=1}^m X_{i,t}^b$ for product B. In Table B1 we present the parameter values that are the same for all of the experiments for Scenario B.

	C_v^a	C_v^b	C_r^a	C_r^b	ρ	γ	ϵ
Value	0.5	0.5	1.0	1.0	0.5	1.0	1×10^{-4}

Table B1: Parameter values that are consistent for all of the experiments for Scenario B.

The stochastic element of the transition is the number of products of each type issued, (H^a, H^b). The number of units of product B issued only depends on the demand for product B and the total stock of product B, but the number of units of product A that are issued depends on the demand for product A, the total stock of product A, and any excess demand for product B for which the customer is willing to accept product A.

Let the demand for product A be D^a, the demand for product B be D^b, the excess demand for product B where the customer is willing to accept product A be D^u and the total demand for product A including any substitution be $D^z = D^a + D^u$. To calculate the probability of a combination $\omega = (h^a, h^b)$ given a particular state s, we consider five possible cases:

$$\begin{aligned}
\mathrm{Prob}\left(\Omega = \omega | S = s, A = a\right) &= P\left(\Omega = \left(h^a, h^b\right)|S = s\right) \\
&= P\left(H^a = h^a, H^b = h^b | S = s\right) \\
&= \begin{cases} 0, & \text{if } h^a > I^a \text{ or } h^b > I^b \\ P(D^a = h^a)P(D^b = h^b), & \text{if } h^a < I^a \text{ and } h^b < I^b \\ P(D^a \geq I^a)P(D^b = h^b), & \text{if } h^a = I^a \text{ and } h^b < I^b \\ P(D^z = h^a | S = s)P(D^b \geq I^b), & \text{if } h^a < I^a \text{ and } h^b = I^b \\ P(D^z \geq I^a)P(D^b \geq I^b), & \text{if } h^a = I^a \text{ and } h^b = I^b \end{cases} \\
&= \begin{cases} 0, & \text{if } h^a > I^a \text{ or } h^b > I^b \\ P(h^a; \mu^a)P(h^b; \mu^b), & \text{if } h^a < I^a \text{ and } h^b < I^b \\ \left[1 - F\left(I^a - 1; \mu^a\right)\right] P(h^b; \mu^b), & \text{if } h^a = I^a \text{ and } h^b < I^b \\ P(D^z = h^a | S = s)\left[1 - \left(F(I^b - 1; \mu^b\right)\right], & \text{if } h^a < I^a \text{ and } h^b = I^b \\ \left[1 - \sum_{d=0}^{I^a - 1} P(D^z = d | S = s)\right]\left[1 - \left(F(I^b - 1; \mu^b\right)\right], & \text{if } h^a = I^a \text{ and } h^b = I^b \end{cases}
\end{aligned} \tag{B.3}$$

For the fourth and fifth cases there may be substitution, and therefore we need to consider the distribution of the total demand for product A and the distribution of the demand for substitution:

$$
\begin{aligned}
P(D^z = d^z | S = s) &= P(D^z = d^z | I^b = y) \\
&= \sum_{k=0}^{d^z} P(D^a = k) P(D^u = d^z - k | I^b = y, D^b \geq y) \\
&= \sum_{k=0}^{d^z} P(k; \mu^a) P(D^u = d^z - k | I^b = y, D^b \geq y)
\end{aligned} \tag{B.4}
$$

$$
\begin{aligned}
P(D^u = d^u | I^b = y, D^b \geq y) &= \begin{cases} \sum_{c=0}^{\infty} P(D^b = c + y)(1-\rho)^c, & \text{if } d^u = 0 \\ \sum_{c=d^u}^{\infty} P(D^b = c + y) P(d^u; c, \rho), & \text{if } d^u > 0 \end{cases} \\
&= \begin{cases} \sum_{c=0}^{\infty} P(c + y; \mu^b)(1-\rho)^c, & \text{if } d^u = 0 \\ \sum_{c=d^u}^{\infty} P(c + y; \mu^b) P(d^u; c, \rho), & \text{if } d^u > 0 \end{cases}
\end{aligned} \tag{B.5}
$$

where $P(x; c, \rho)$ is a binomial probability mass function representing the probability that there are x units of excess demand for product B willing to accept product A out of a total of c units of excess demand for product B and the probability of being willing to accept the substitution is ρ. $P(x; \mu^a)$ and $P(x; \mu^b)$ are the probability mass functions of independent Poisson distributions for the daily demand of product A and B parameterised by mean daily demands μ^a and μ^b respectively, and $F(x; \mu^a)$ and $F(x; \mu^b)$ are the corresponding cumulative distribution functions.

We calculated the values of $P(D^u = d^u | I^b = y, D^b \geq y)$ and $P(D^z = d^z | S = s)$, for $d^u \in \{0, 1, ..., D_{\max}\}$ and $d^z \in \{0, 1, ..., D_{\max}\}$, where $D_{\max} = \left((m \max(A^a_{\max}, A^b_{\max})) + 2 \right)$, at the start of value iteration following the MATLAB implementation of Hendrix et al. (2019).

The reward function comprises two components which can be different for each product: a variable ordering cost per unit (C^a_v, C^b_v) and revenue per unit sold (C^a_r, C^b_r). The single step reward after taking action A_t in state S_t with $\Omega_t = \left(H^a_t, H^b_t \right)$ is:

$$
R_{t+1} = -\left(C^a_v A^a_t + C^a_v A^b_t \right) + \left(C^a_r H^a_t + C^b_r H^b_t \right) \tag{B.6}
$$

Equation B.7 shows how the elements of $\underline{X}^a_t$ and $\underline{X}^b_t$ are updated following a FIFO issuing policy.

$$
\begin{aligned}
X^a_{j,t+1} &= X^a_{j+1,t} - \left[H^a_t - \sum_{k=1}^{j} X^a_{k,t} \right]^+ \quad && \forall j \in \{1, 2, ..., m-1\} \\
X^a_{m,t+1} &= A^a_t \\
X^b_{j,t+1} &= X^b_{j+1,t} - \left[H^b_t - \sum_{k=1}^{j} X^b_{k,t} \right]^+ \quad && \forall j \in \{1, 2, ..., m-1\} \\
X^b_{m,t+1} &= A^b_t
\end{aligned} \tag{B.7}
$$

The maximum order quantities for value iteration, $A^a_{\max}$ and $A^b_{\max}$ are calculated independently for each product following the newsvendor model (Snyder & Shen, 2019):

$$
A^k_{\max} = \left\lceil F^{-1} \left(\frac{C^k_r - C^k_v}{C^k_r}; m\mu^k \right) \right\rceil^+, \quad \forall k \in \{a, b\} \tag{B.8}
$$

where $F(x; m\mu^k)$ is the cumulative distribution function of a Poisson distribution parameterised by $m\mu^k$ and μ^k is the mean daily demand for product k.

The maximum order quantities reported for experiments P1 to P4 in Table 3 of Ortega et al. (2019) are not consistent with the number of states reported in that table. We have assumed that they instead represent the number of actions (one higher than the maximum order quantity, due to the possibility of ordering zero units) as this is consistent with the number of states reported, with Table 1 of Ortega et al. (2019) and, for experiment P1, with the corresponding experiment in Hendrix et al. (2019).

The initial estimate for the value function is the expected one-step ahead sales revenue:

$$V_0(s) = \sum_{h^a=0}^{I_t^a} \sum_{h^b=0}^{I_t^b} P(H^a = h^a, H^b = h^b | S = s)(h^a C_r^a + h^b C_r^b) \tag{B.9}$$

We used the same convergence test as Hendrix et al. (2019), evaluating:

$$\max_{s \in \mathbb{S}} [V_i(s) - V_{i-1}(s)] - \min_{s \in \mathbb{S}} [V_i(s) - V_{i-1}(s)] < \epsilon \tag{B.10}$$

after each iteration. The inequality tests for the convergence of the change in value for each state. When the value of each state is changing by the same amount, the best action for each state will not change and therefore the estimate of the optimal policy is stable. We saved a checkpoint after every iteration.

B.2 Additional results

We present additional results for the experimental settings from Hendrix et al. (2019) in Table B2 and for the experimental settings from Ortega et al. (2019) in Tables B3 and B4. Table B3 follows the same format as Table 4. In Tables B2 and B4 we present the best combination of order-up-to level parameters $\left(\mathbb{S}^a, \mathbb{S}^b\right)_{\text{best}}$ fit using simulation optimization and the mean and standard deviation of three KPIs calculated over 10,000 evaluation rollouts for each policy. Demand for product B was considered to be satisfied for the purposes of calculating the service level if filled by product A when substitution was acceptable.

The only difference between experiment 1 from Hendrix et al. (2019) and experiment P1 from Ortega et al. (2019) is that value iteration was run for 100 iterations for experiment P1: more than were required for convergence. The best parameters for the modified base-stock policy and the KPIs from evaluating the policies are the same for these experiments, as we would expect.

m	Exp	Product	$\mathtt{S}_{\text{best}}$	**Service level (%)** VI	SO	**Wastage (%)** VI	SO	**Holding (units)** VI	SO
2	1	A	13	95.5 ± 0.8	95.2 ± 0.8	6.0 ± 1.0	6.3 ± 1.0	2.7 ± 0.1	2.7 ± 0.1
		B	12	94.9 ± 0.8	95.5 ± 0.7	4.2 ± 0.8	5.3 ± 0.9	2.1 ± 0.1	2.3 ± 0.1
	2	A	18	96.9 ± 0.6	96.6 ± 0.6	4.2 ± 0.8	4.4 ± 0.7	3.7 ± 0.2	3.6 ± 0.2
		B	7	91.5 ± 1.1	92.5 ± 1.0	6.5 ± 1.2	8.3 ± 1.3	1.2 ± 0.1	1.3 ± 0.1
3	1	A	15	98.3 ± 0.5	98.3 ± 0.5	2.2 ± 0.6	2.4 ± 0.6	4.9 ± 0.2	4.8 ± 0.2
		B	14	98.4 ± 0.4	98.4 ± 0.4	1.5 ± 0.5	1.7 ± 0.5	4.1 ± 0.2	4.1 ± 0.2
	2	A	21	99.1 ± 0.3	99.2 ± 0.3	1.2 ± 0.4	1.3 ± 0.4	6.7 ± 0.3	6.7 ± 0.2
		B	8	96.6 ± 0.7	96.1 ± 0.7	3.3 ± 0.9	3.2 ± 0.8	2.4 ± 0.1	2.3 ± 0.1
	3	A	15	98.3 ± 0.5	98.3 ± 0.5	2.2 ± 0.6	2.4 ± 0.6	4.9 ± 0.2	4.8 ± 0.2
		B	14	98.4 ± 0.4	98.4 ± 0.4	1.5 ± 0.5	1.7 ± 0.5	4.1 ± 0.2	4.1 ± 0.2
	4	A	21	99.1 ± 0.3	99.2 ± 0.3	1.2 ± 0.4	1.3 ± 0.4	6.6 ± 0.3	6.7 ± 0.2
		B	8	96.6 ± 0.7	96.1 ± 0.7	3.3 ± 0.9	3.2 ± 0.8	2.4 ± 0.1	2.3 ± 0.1

Table B2: The best combination of order-up-to levels $\left(\mathtt{S}^a, \mathtt{S}^b\right)_{\text{best}}$, fit using simulation optimization, and KPIs for policies fit using value iteration (VI) and simulation optimization (SO) for all of the experimental settings for Scenario B from Hendrix et al. (2019)

m	Exp	μ^a	μ^b	$A^a_{\max}$	$A^b_{\max}$	$\lvert\mathbb{S}\rvert$	$\lvert\mathbb{A}\rvert$	$\lvert\Omega\rvert$	**Value iteration** WT (s)	Return	**Simulation optimization** WT (s)	Return	Optimality gap (%)
2	P1	5	5	10	10	14,641	121	441	11	1,644 ± 33	25	1,632 ± 34	0.70
	P2	5	6	10	12	20,449	143	525	18	1,826 ± 35	31	1,816 ± 34	0.58
	P3	6	6	12	12	28,561	169	625	27	2,011 ± 36	31	2,000 ± 37	0.55
	P4	7	7	13	13	38,416	196	729	56	2,379 ± 39	29	2,368 ± 40	0.46

Abbreviations: m: maximum useful life, μ^p: mean demand for product p, $A^p_{\max}$: maximum order quantity for product p, $\lvert\mathbb{S}\rvert$: number of possible states, $\lvert\mathbb{A}\rvert$: number of possible actions, $\lvert\Omega\rvert$: number of possible realisations of stochastic elements in a transition, WT: wall time.

Table B3: Our results on Scenario B for all of the experimental settings used by Ortega et al. (2019) to test their GPU-accelerated approach. Value iteration was run for 100 iterations for each experiment, more than was required for convergence. Aside from this, experiment P1 is the same as experiment 1 with $m = 2$ in Table 4. The longest wall time, for value iteration on experiment P4, is approximately one minute. Value iteration was tractable for all of these settings in the original study but wall times using our method are at least six times faster than those reported by Ortega et al. (2019). This improvement may be at least partially attributable to hardware differences.

m	Exp	Product	$\mathtt{S}_{\text{best}}$	**Service level (%)** VI	SO	**Wastage (%)** VI	SO	**Holding (units)** VI	SO
2	P1	A	13	95.5 ± 0.8	95.2 ± 0.8	6.0 ± 1.0	6.3 ± 1.0	2.7 ± 0.1	2.7 ± 0.1
		B	12	94.9 ± 0.8	95.5 ± 0.7	4.2 ± 0.8	5.3 ± 0.9	2.1 ± 0.1	2.3 ± 0.1
	P2	A	13	95.5 ± 0.8	95.1 ± 0.8	6.0 ± 1.0	6.2 ± 0.9	2.7 ± 0.1	2.6 ± 0.1
		B	14	95.9 ± 0.6	95.5 ± 0.7	3.5 ± 0.7	3.7 ± 0.7	2.5 ± 0.1	2.5 ± 0.1
	P3	A	16	96.2 ± 0.7	96.8 ± 0.6	4.9 ± 0.9	6.0 ± 0.9	3.2 ± 0.2	3.4 ± 0.1
		B	14	95.9 ± 0.6	95.8 ± 0.6	3.5 ± 0.7	3.7 ± 0.7	2.5 ± 0.1	2.5 ± 0.1
	P4	A	18	96.8 ± 0.6	96.7 ± 0.6	4.2 ± 0.8	4.5 ± 0.8	3.7 ± 0.2	3.7 ± 0.2
		B	17	96.5 ± 0.6	97.1 ± 0.5	2.9 ± 0.6	3.8 ± 0.7	2.9 ± 0.2	3.2 ± 0.1

Table B4: The best combination of order-up-to levels $\left(\mathtt{S}^a, \mathtt{S}^b\right)_{\text{best}}$, fit using simulation optimization, and KPIs for policies fit using value iteration (VI) and simulation optimization (SO) for all of the experimental settings for Scenario B from Ortega et al. (2019)

C Additional information for Scenario C

C.1 Scenario description

In this section we recast the problem formulated by Mirjalili (2022) into a consistent notation used for all three of the scenarios.

The state of the environment, S_t, comprises two components: $\tau \in \{0, 1, ..., 6\}$, representing the day of the week, and the units in stock at the start of the day $\underline{\mathrm{X}_t} = [X_{m-1,t}, X_{m-2,t}, ..., X_{1,t}]$. The lead time, L, is always zero which means that the units ordered on day t are received before any demand arises on day t. There are therefore only $m-1$ elements in $\underline{\mathrm{X}_t}$ and a total of m elements, including τ, in S_t.

In the previous problems, the maximum value of each element of $\underline{\mathrm{X}_t}$ was $A_{\max}$, because all units arrived with the same remaining useful life. All units received in the same period would be in the same element of $\underline{\mathrm{X}_t}$. In this scenario, the remaining useful life on arrival is stochastic and therefore, depending on the policy, a series of orders could be received such that an element of $\underline{\mathrm{X}_t}$ would exceed $A_{\max}$. We assume there is a maximum capacity of $A_{\max}$ for stock of each possible value of remaining useful life. Units received in excess of this limit are not accepted at the point of delivery. The total number of possible states is therefore $7 \times (A_{\max} + 1)^{m-1}$. The entries in $\underline{\mathrm{X}_t}$ are ordered by ascending age: the first element represents stock with $m-1$ days before expiry, and the last element is the stock that will expire at the end of day t. In Table C1 we present the parameter values that are the same for all of the experiments for Scenario C.

	$D_{\max}$	$A_{\max}$	C_f	C_h	C_s	C_w	γ	ϵ
Value	20	20	10	1	20	5	0.95	1×10^{-4}

Table C1: Parameter values that are consistent for all of the experiments for Scenario C.

The stochastic elements in the transition are the daily demand D, and the age profile of the units received: $\underline{\mathrm{Y}} = [Y_m, Y_{m-1}, ..., Y_1]$.

The probability of a given random outcome ω is the product of the probability of the demand given the state, and the probability of receiving units with a specific age profile given the action:

$$\begin{aligned} \text{Prob}\left(\Omega = \omega | S = s, A = a\right) &= P\left(\Omega = \left(d, \underline{\mathrm{y}}\right) | S = s, A = a\right) \\ &= P\left(D = d, \underline{\mathrm{Y}} = \underline{\mathrm{y}} | S = s, A = a\right) \\ &= P\left(D = d | S = s\right) P\left(\underline{\mathrm{Y}} = \underline{\mathrm{y}} | A = a\right) \end{aligned} \tag{C.1}$$

Demand is modelled by truncated negative binomial distributions, one for each day of the week. The demand distribution therefore only depends on the weekday element of the state. The negative binomial distribution models the number of failures, x, in a series of repeated Bernoulli trials before achieving a specified number of successes. The probability that daily demand is equal to d on weekday τ is:

$$P(D = d | S = s) = \begin{cases} P(d; n^\tau, \delta^\tau), & \text{if } d \in \{0, 1, ..., D_{\max} - 1\} \\ 1 - F(D_{\max} - 1; n^\tau, \delta^\tau), & \text{if } d = D_{\max} \end{cases} \tag{C.2}$$

where $P(x; n^\tau, \delta^\tau)$ is the probability mass function of a negative binomial distribution parameterised by a target number of successes n^τ and a mean δ^τ for weekday τ and $F(x; n^\tau, \delta^\tau)$ is the corresponding cumulative distribution function. The probability of success in an individual Bernoulli trial is $p^\tau = \frac{n^\tau}{n^\tau + \delta^\tau}$. The parameters for each day of the week are set out in Table C2.

τ	0	1	2	3	4	5	6
n^τ	3.5	11.0	7.2	11.1	5.9	5.5	2.2
δ^τ	5.7	6.9	6.5	6.2	5.8	3.3	3.4

Table C2: Parameters of the demand distribution for each weekday from Monday ($\tau = 0$) to Sunday ($\tau = 6$)

The remaining useful life of units on arrival is modelled by a multinomial distribution with a number of trials equal to the order quantity a and a number of events equal to the maximum useful life m. The probability mass function for the distribution is:

$$\begin{aligned} P(\underline{\mathrm{Y}} = \underline{\mathrm{y}}|A = a) &= P(Y_m = y_m, ..., Y_1 = y_1|A = a) \\ &= \begin{cases} \frac{a!}{y_m! y_{m-1}! ... y_1!} p_m(a)^{y_m} p_{m-1}(a)^{y_{m-1}} ... p_1(a)^{y_1}, & \text{if } a = \sum_{i=1}^m y_i \\ 0, & \text{if } a \neq \sum_{i=1}^m y_i \end{cases} \end{aligned} \tag{C.3}$$

The parameters of the multinomial distribution are modelled by an affine function of the order quantity a:

$$\log\left(\frac{p_k(a)}{p_1(a)}\right) = c_0^k + c_1^k a, \quad \forall k \in \{2, 3, .., m\} \tag{C.4}$$

If the distribution of remaining useful life on arrival does not depend on order quantity, and therefore the uncertainty is exogenous, $c_1^k = 0 \quad \forall k \in \{2, 3, .., m\}$. The values of c_0^k and c_1^k for our experiments are set out in Table C3. These represent a subset of the experiments run by Mirjalili (2022). The parameters for the two experiments where $m = 5$ were determined by Mirjalili (2022) by fitting multinomial logistic regression models to observed data from a hospital system in Ontario, Canada.

m	Exp	c_0^2	c_0^3	c_0^4	c_0^5	c_0^6	c_0^6	c_0^8	c_1^2	c_1^3	c_1^4	c_1^5	c_1^6	c_1^7	c_1^8
3	1	1.0	0.5												
	2	1.0	0.5						0.40	0.80					
5	1	1.6	2.6	2.8	1.6										
	2	1.9	3.1	3.1	2.5				-0.03	-0.06	-0.03	-0.09			
8	1	0.8	1.4	1.9	2.3	1.7	1.2	0.8							
	2	0.8	1.4	1.9	2.3	1.7	1.2	0.8	-0.03	-0.04	-0.05	-0.06	-0.07	-0.08	-0.09

Table C3: Parameters for the affine function used to model the parameters of the multinomial distribution of remaining useful life on arrival for each experiment. These are a subset of the experiments described by Mirjalili (2022)

.

The reward function comprises four components: a holding cost per unit in stock at the end of the period (C_h), a shortage cost per unit of unmet demand (C_s), a wastage cost per unit that perishes at the end of the period (C_w) and a fixed ordering cost which is incurred when $A_t > 0$ (C_f). The single-step reward function after taking action A_t in state $S_t = \left(\tau_t, \underline{\mathrm{X}_t}\right)$, and observing $\Omega_t = (D_t, \underline{\mathrm{Y}_t})$ is

$$R_{t+1} = -C_f \mathbb{1}_{A_t>0} - C_h \left[Y_{m,t} + \sum_{i=1}^{m-1} \min\left(X_{i,t} + Y_{i,t}, A_{\max}\right) - D_t \right]^+$$
$$- C_s \left[D_t - Y_{m,t} - \sum_{i=1}^{m-1} \min\left(X_{i,t} + Y_{i,t}, A_{\max}\right) \right]^+ - C_w \left[\min\left(X_{1,t} + Y_{1,t}, A_{\max}\right) - D_t\right]^+ \quad \text{(C.5)}$$

Equation C.6 shows how the elements of the state are updated, following a OUFO policy:

$$\tau_{t+1} = (\tau_t + 1) \mod 7 \quad \text{(C.6)}$$
$$X_{j,t+1} = \left[\min\left(X_{j+1,t} + Y_{j+1,t}, A_{\max}\right) - \left[D_t - \sum_{k=1}^{j} \min\left(X_{k,t} + Y_{k,t}, A_{\max}\right) \right]^+ \right]^+ \quad \forall j \in \{1, ..., m-2\}$$
$$X_{m-1,t+1} = \left[Y_{m,t} - \left[D_t - \sum_{k=1}^{m-1} \min\left(X_{k,t} + Y_{k,t}, A_{\max}\right) \right]^+ \right]^+$$

The scenario is an infinite horizon MDP with a discount factor and periodicity because the demand depends on the day of the week. We take advantage of the periodicity of the problem, and use a convergence test based on those described by Su & Deininger (1972). Performing this convergence test requires retaining at least the last seven (as this is the periodicity) estimates of the value function, and we can only test for convergence after we have run at least seven iterations. We tested the following inequality at the end of each iteration once $i \geq 7$:

$$\Delta_{\max,i} = \max_{s \in \mathbb{S}} \left[\sum_{j=0}^{6} \frac{1}{\gamma^{i-j-1}} \left(V_{i-j}(s) - V_{i-j-1}(s)\right) \right] \quad \text{(C.7)}$$
$$\Delta_{\min,i} = \min_{s \in \mathbb{S}} \left[\sum_{j=0}^{6} \frac{1}{\gamma^{i-j-1}} \left(V_{i-j}(s) - V_{i-j-1}(s)\right) \right] \quad \text{(C.8)}$$
$$\Delta_{\max,i} - \Delta_{\min,i} \leq 2\epsilon \min\left[|\Delta_{\max,i}|, |\Delta_{\min,i}|\right] \quad \text{(C.9)}$$

When the inequality is met, the additional undiscounted reward being added to each state in one whole cycle (one week) is approximately the same, subject to our confidence level. In turn this means that for each weekday, every state is being increased by the same amount and therefore the best action for each state will not change. We therefore terminated value iteration when the inequality was met. If there were no discounting, and so $\gamma = 1$, the term in the square brackets would be equal to $V_i(s) - V_{i-7}(s)$: the total change in value from one cycle (in this case, one week). This convergence test relies on checkpoints from previous iterations, and we therefore saved a checkpoint every iteration.

C.2 Additional results

In Table C4 we present the best combination of parameters for the heuristic policy fit using simulation optimization. In Table C5 we present the mean and standard deviation of three KPIs calculated over 10,000 evaluation rollouts for each policy. For consistency with the calculation of the reward function in Mirjalili (2022) the holding KPI includes the units that will expire at the end of the current day. These units are excluded from the calculation of the stock holding at the end of the day in the other scenarios

			Weekday τ						
m	Exp	Parameter	0	1	2	3	4	5	6
3	1	$\mathtt{S}_{\text{best}}$	13	12	14	11	11	8	7
		$\mathtt{s}_{\text{best}}$	6	7	7	6	6	3	3
	2	$\mathtt{S}_{\text{best}}$	14	14	15	13	12	9	9
		$\mathtt{s}_{\text{best}}$	7	7	7	7	6	3	4
5	1	$\mathtt{S}_{\text{best}}$	16	17	16	13	13	10	14
		$\mathtt{s}_{\text{best}}$	7	8	8	7	7	3	3
	2	$\mathtt{S}_{\text{best}}$	17	16	16	13	13	11	14
		$\mathtt{s}_{\text{best}}$	7	7	9	8	8	3	4
8	1	$\mathtt{S}_{\text{best}}$	19	15	18	18	14	13	16
		$\mathtt{s}_{\text{best}}$	8	8	8	7	8	3	4
	2	$\mathtt{S}_{\text{best}}$	18	18	16	15	14	11	15
		$\mathtt{s}_{\text{best}}$	9	8	9	7	7	3	4

Table C4: The best combination of parameters for the heuristic policy $\left(\left(\mathtt{s}^0, \mathtt{S}^0\right), ..., \left(\mathtt{s}^6, \mathtt{S}^6\right)\right)_{\text{best}}$ fit using simulation optimization for each of our experiments for Scenario C, a subset of the experiments run by Mirjalili (2022).

		Service level (%)		Wastage (%)		Holding (units)	
m	Exp	VI	SO	VI	SO	VI	SO
3	1	95.3 ± 0.9	95.3 ± 0.9	12.6 ± 1.3	12.6 ± 1.4	4.9 ± 0.1	4.9 ± 0.1
	2	96.6 ± 0.8	96.2 ± 0.8	7.0 ± 1.1	7.2 ± 1.1	5.8 ± 0.1	5.7 ± 0.1
5	1	97.4 ± 0.7	97.0 ± 0.7	3.2 ± 0.8	3.0 ± 0.7	6.8 ± 0.1	6.7 ± 0.1
	2	97.4 ± 0.7	97.5 ± 0.7	3.1 ± 0.7	3.4 ± 0.8	6.8 ± 0.1	7.0 ± 0.2
8	1	—	97.9 ± 0.6	—	0.7 ± 0.3	—	8.0 ± 0.2
	2	—	97.7 ± 0.6	—	1.0 ± 0.4	—	7.5 ± 0.2

Table C5: KPIs for policies fit using value iteration (VI) and simulation optimization (SO) for each of our experiments for Scenario C, a subset of the experiments run by Mirjalili (2022). Value iteration was not feasible when $m = 8$.

D Notation

In Table D1 we summarise the notation we have used to recast the problems described in Scenarios A, B and C. In Scenario B we use a superscript a for product A and b for product B if a variable is product-specific. In Scenario C we use a superscript τ (or weekday index from 0 to 6 representing Monday to Sunday, respectively) if a variable is weekday-specific. We drop the subscript t in some contexts where elements from different days do not feature.

Markov decision process	$\mathbb{S}$	Set of possible states
	S_t	State observed at the start of day t
	s	A specific element of $\mathbb{S}$
	$\mathbb{A}$	Set of possible actions
	A_t	Action taken at the start of day t after observing S_t
	a	A specific element of $\mathbb{A}$
	$\mathbb{\Psi}$	Set of possible rewards
	R_t	Reward received when state S_t is observed
	r	A specific element of $\mathbb{\Psi}$
	$\mathbb{\Omega}$	Set of possible realisations of stochastic elements in a transition
	Ω_t	Realisation of the stochastic elements in the transition between S_t and S_{t+1}
	ω	A specific element of $\mathbb{\Omega}$
	γ	Discount factor
	G_t	Return, the discounted sum of rewards received after taking action A_t
	$\pi(s)$	Policy, a function mapping a state to an action
	$\pi^*(s)$	Optimal policy, policy with the maximum expected return from every state
	$V^\pi(s)$	Value function, expected return starting in state s and following policy π
	$Q^\pi(s,a)$	State-action value function, expected return taking action a in state s and following policy π thereafter
	$T(s,a,\omega)$	Deterministic transition function
Reward function components	C_v	Variable ordering cost per unit
	C_f	Fixed ordering cost
	C_w	Wastage cost per unit
	C_s	Shortage cost per unit
	C_h	Holding cost per unit
	C_r	Revenue per unit
Value iteration	ϵ	Tolerance for convergence test
Heuristic policy parameters	S	Order-up-to level
	s	Reorder point
Inventory control	L	Lead time
	m	Maximum useful life
	D_t	Demand on day t
	$D_{\max}$	Maximum daily demand
	$A_{\max}$	Maximum daily order quantity
	$\underline{\mathrm{X}_t}$	Vector of stock on hand at the start of day t, ordered by ascending age
	$X_{i,t}$	Element of $\underline{\mathrm{X}_t}$ with i days of remaining useful life at the start of day t
	X_t	Total stock on hand at the start of day t
	I_t	Total stock on hand and in transit at the start of day t
Scenario A	$\underline{\mathrm{O}_t}$	Vector of stock in transit at the start of day t, ordered by ascending age
	$O_{i,t}$	Element of $\underline{\mathrm{O}_t}$ that will arrive in i periods at the start of day t
	O_t	Total stock in transit at the start of day t
	W_t	Number of units that expire at the end of day t
	μ	Mean of the gamma distribution for daily demand
	$\frac{\mu}{\sigma}$	Coefficient of variation of the gamma distribution for daily demand
Scenario B	H_t	Number of units of a product issued to fill demand arising on day t
	D_t^u	Excess demand for product B willing to accept product A on day t
	D_t^z	Total demand for product A, including any substitution, on day t
	μ	Mean of the Poisson distribution for daily demand
	ρ	Probability a customer is willing to accept product A if product B is out of stock
Scenario C	τ_t	Day of the week for day t
	$\underline{\mathrm{Y}_t}$	Vector of stock received to fill order A_t, ordered by ascending age
	$Y_{i,t}$	Element of $\underline{\mathrm{Y}_t}$ with i periods of remaining useful life on arrival
	Y_t	Total stock received on day t
	n	Target number of successes for the negative binomial distribution for daily demand
	δ	Mean of the negative binomial distribution for daily demand
	c_0^k	Log-odds ratio of receiving a unit with a remaining useful life of k days versus 1 day when the uncertainty is exogenous.
	c_1^k	Increase in the log-odds ratio of receiving a unit with a remaining useful life of k days versus 1 day for each unit ordered when the uncertainty is endogenous

Table D1: Summary of notation used in this study.
